# Clinical Anatomy of the Descending Genicular Artery and Genicular Arterial Network for Embolization and Reconstructive Surgery: A Systematic Review and Evidence Map with Targeted Meta-Analysis

**DOI:** 10.3390/diagnostics16213642

**Published:** 2026-11-04

**Authors:** Alejandro Bruna-Mejias, Valentina Perez-Lira, Diego Santander-Chavez, Mathis Miranda-Schoen, Juan José Valenzuela-Fuenzalida, Alvaro Bruna-López, Christopher Blackwood-Espinoza, Gustavo Oyanedel-Amaro, Gloria Cifuentes-Suazo, Mathias Orellana-Donoso, Juan J. Cabezas-Salgado, José E. León-Rojas, Yasna Jeria-Pizarro, Gustavo Vega-Fernández, Juan Sanchis-Gimeno

**Affiliations:** 1 Department of Sciences and Geography, Faculty of Natural and Exact Sciences, Universidad de Playa Ancha, Valparaíso 2360072, Chile; alejandro.bruna@unab.cl; 2 School of Medicine, Faculty of Medicine, Universidad Andrés Bello, Viña del Mar 2520000, Chile; v.prezlira@uandresbello.edu (V.P.-L.); d.santanderchvez@uandresbello.edu (D.S.-C.); m.mirandaschoen@uandresbello.edu (M.M.-S.); juan.kine.2015@gmail.com (J.J.V.-F.); 3 Department of Chemical and Biological Sciences, Faculty of Health Sciences, Universidad Bernardo O’Higgins, Santiago 8370993, Chile; 4 Emergency Department, Magallanes Health Service, Hospital Clínico de Magallanes, Punta Arenas 6200000, Chile; alvaro.bruna97@gmail.com; 5 School of Medicine, Universidad de Magallanes, Punta Arenas 6200000, Chile; 6 School of Health Sciences, Universidad Viña del Mar, Viña del Mar 2520000, Chile; christopher.blackwood@docente.uvm.cl; 7 Faculty of Health and Social Sciences, University of the Americas, Santiago 8370040, Chile; g.oyanedelamaro@gmail.com; 8 Dentistry Program, Faculty of Medicine, Universidad Católica de la Santísima Concepción, Concepción 4090541, Chile; gbcifuentess@gmail.com; 9 School of Medicine, Universidad Finis Terrae, Santiago 7501015, Chile; mathor94@gmail.com; 10 Faculty of Medicine and Science, Universidad San Sebastián, Santiago 7510157, Chile; 11 Faculty of Medicine, Universidad Católica del Maule, Talca 3460000, Chile; jcabezas@ucm.cl; 12 Cerebro, Emoción y Conducta (CEC) Research Group, School of Medicine, Universidad de las Américas (UDLA), Quito 170521, Ecuador; 13 Faculty of Health Sciences, Universidad Autónoma de Chile, Santiago 8910060, Chile; yasnajeria@gmail.com; 14 Laboratory of Epidemiology and Morphological Sciences, Institute of Biology, Pontificia Universidad Católica de Valparaíso, Valparaíso 2373223, Chile; gustavo.vega@pucv.cl; 15 GIAVAL Research Group, Department of Anatomy and Human Embryology, Faculty of Medicine, University of Valencia, 46001 Valencia, Spain; juan.sanchis@uv.es

**Keywords:** descending genicular artery, genicular artery embolization, cone-beam CT, digital subtraction angiography, clinical anatomy, medial femoral condyle flap, saphenous artery, systematic review, meta-analysis

## Abstract

**Background/Objectives:** The descending genicular artery (DGA) is relevant to genicular artery embolization (GAE), medial femoral condyle and perforator flaps, recipient-vessel selection, and vascular complications around the knee. This review integrated cadaveric, imaging, procedural, reconstructive, and safety evidence and examined whether bilateral cadaveric sampling affected pooled estimates. **Methods:** PubMed/MEDLINE, Scopus, Web of Science/SCIE, CENTRAL/Ovid, and LILACS/BVS were searched through 10 June 2026, followed by a targeted update on 18 August 2026. Evidence was stratified by design; anatomical prevalence was not pooled with in vivo visualization. Anatomical studies were appraised using an AQUA-adapted framework, imaging studies using the JBI analytical cross-sectional checklist, reconstructive series using the JBI case-series checklist, and case reports using the JBI case-report checklist. Compatible outcomes were synthesized with REML random-effects models and Knapp–Hartung confidence intervals. **Results:** Sixty-five primary studies were included, and 22 contributed to at least one quantitative synthesis. Strict cadaveric DGA presence was 91.6% (95% CI 86.6–94.8; I^2^ = 30.3%); a worst-case cluster-adjusted sensitivity estimate accounting for bilateral specimens was 89.7% (84.7–93.2%). Femoral/superficial femoral origin was 92.4% (89.2–94.7), popliteal origin 3.2% (2.2–4.6), and an articular/osteoarticular branch 90.3% (84.1–94.2). In vivo DGA visualization during GAE-related imaging was 95.9% (93.1–97.6) and was interpreted separately from anatomical prevalence. Reconstructive and safety evidence was predominantly derived from small series and case reports. **Conclusions:** The DGA is usually identifiable and generally arises from the femoral arterial system, but branch-level variability remains clinically important. Cadaveric anatomy and procedural imaging are complementary. The anatomical findings may inform GAE and reconstructive planning, whereas clinical safety and reconstructive conclusions require confirmation in larger cohorts.

## 1. Introduction

The descending genicular artery (DGA) is a clinically important arterial axis of the distal medial thigh and knee. It usually arises from the distal femoral or superficial femoral artery and contributes to the medial femoral condyle, periarticular soft tissues, articular and osteoarticular territories, and the saphenous vascular axis. Cadaveric studies consistently identify the vessel but also document variation in origin, dominance, branching, and its relationship with the superior medial genicular artery (SMGA) [1,2,3,4]. The anatomical relationships synthesized in this review are summarized in Figure 1.

Interest in the DGA has increased with the expansion of genicular artery embolization (GAE) for symptomatic knee osteoarthritis and selected post-arthroplasty pain syndromes. GAE requires selective identification of target vessels, distinction between articular and superficial branches, and recognition of anastomotic pathways that may influence embolization efficacy and non-target risk. Angiography, cone-beam computed tomography (CBCT), digital subtraction angiography (DSA), computed tomography angiography (CTA), and Doppler imaging provide in vivo information that complements, but does not replace, cadaveric anatomy [5,6,7,8,9,10]. Recent technical and anatomical reviews have likewise emphasized the need for branch-level mapping and standardized vascular nomenclature [11,12].

The same arterial system is relevant to reconstructive surgery. The DGA and its branches support medial femoral condyle corticoperiosteal, osteocutaneous, and chimeric flaps, DGA perforator flaps, saphenous-axis flaps, and recipient-vessel strategies around the knee [13,14,15,16,17,18,19,20,21,22,23,24,25,26,27,28,29]. Recent technical reports additionally demonstrate the use of the DGA as an access or recipient route in selected vascular and reconstructive settings [30,31,32].

Safety-relevant reports provide a further reason to synthesize this literature. DGA and related genicular-branch injuries have been described after arthroscopy, anterior cruciate ligament reconstruction, transient patellar dislocation, and post-arthroplasty interventions, frequently presenting as pseudoaneurysm, active bleeding, or hematoma [33,34,35,36,37]. A recent supra-articular branch aneurysm report further illustrates that genicular vascular lesions may mimic soft-tissue tumors and require careful imaging assessment [38].

Despite growing clinical relevance, the evidence remains fragmented across anatomical dissection, angiographic series, CBCT/DSA studies, CTA/Doppler investigations, reconstructive case series, and safety reports. Cadaveric presence, in vivo visualization, and procedural target use represent different constructs and should not be pooled indiscriminately. This systematic review therefore aimed to synthesize the clinically relevant anatomy of the DGA and genicular arterial network, map interventional and reconstructive implications, and perform targeted meta-analysis only when definitions, denominators, and units of analysis were compatible.

## 2. Methods

### 2.1. Protocol and Registration

This systematic review and evidence map followed PRISMA 2020 reporting principles [39] and a prospectively registered protocol (PROSPERO CRD420261412569; https://www.crd.york.ac.uk/prospero/; accessed on 18 August 2026). The review question, eligibility framework, evidence strata, primary anatomical outcomes, and conditional meta-analysis rules were defined before full extraction. The reviewer-driven unit-of-analysis sensitivity and targeted literature update reported in this revision were analytical clarifications undertaken in response to peer review; they did not alter eligibility criteria or outcomes after inspection of pooled results.

### 2.2. Eligibility Criteria

Eligible evidence comprised human cadaveric dissection or perfusion studies; angiographic, CBCT, DSA, CTA, or Doppler studies reporting vessel-level genicular anatomy; GAE studies with extractable vessel-level information; reconstructive studies involving the DGA or related genicular arteries; and safety reports of DGA or genicular-branch injury. Non-human studies, biomechanical simulations without primary human vascular data, commentaries or letters without original data, reviews without extractable primary data, and clinical GAE efficacy reports lacking anatomical, procedural, or vessel-level information were excluded from primary synthesis. Cadaveric, imaging/procedural, reconstructive, and safety evidence were retained as separate strata.

### 2.3. Information Sources and Search Strategy

PubMed/MEDLINE, Scopus, Web of Science Core Collection/SCIE, CENTRAL via Ovid EBM Reviews, and LILACS/BVS were searched from inception to 10 June 2026 without date or language restrictions. The complete platform-specific strategies, execution dates, and yields are reported in Supplementary Table S1. Citation checking was used for contextual verification and did not add records to the original PRISMA flow. In response to peer review, a targeted update was performed on 18 August 2026 using the core terms ‘descending genicular artery’, ‘genicular artery anatomy’, ‘genicular artery embolization’, ‘genicular artery imaging’, and ‘descending genicular artery flap’. The update identified six post-cutoff publications [11,12,30,31,32,38]: two reviews [11,12] and four original reports [30,31,32,38]. These publications were incorporated into the updated narrative context but were not added to the formal 65-study evidence set or the frozen quantitative datasets because they appeared after the prespecified search cutoff and did not provide data compatible with any prespecified pooled outcome. Their disposition is documented in Supplementary Table S10.

### 2.4. Study Screening and Eligibility Assessment

The protocol specified duplicate independent screening. Each record was assessed by two members of a four-reviewer screening team (A.B.-M., A.B.-L., J.J.V.-F., and G.O.-A.) using predefined categories (include, exclude, unclear/retrieve full text, citation-only, or residual duplicate). Full-text eligibility was likewise assessed in duplicate. Disagreements were resolved by consensus, with senior methodological adjudication by J.S.-G. when required. Reviewers were not blinded to authorship or journal because blinding is not routinely required for systematic review screening. Records were managed and deduplicated in Zotero, and reasons for full-text exclusion were recorded prospectively.

### 2.5. Data Extraction and Evidence Stratification

A standardized extraction form captured bibliographic data, design, preservation or imaging method, sample size, number of cadavers and anatomical units, laterality, unit of analysis, DGA presence and origin, branch pattern, saphenous and articular/osteoarticular branches, morphometry, in vivo visualization, reconstructive application, and safety-relevant vascular events. Data were extracted into a predefined R-ready matrix by one reviewer and independently verified against the source report by a second reviewer; disagreements were resolved by consensus and senior methodological adjudication. Stratum A comprised cadaveric DGA-core anatomy; Stratum B, imaging/procedural evidence; Stratum C, safety reports; Stratum D, reconstructive evidence; and Stratum E, context-only records not counted as primary evidence.

### 2.6. Methodological Quality Assessment

Methodological quality was assessed by design. Cadaveric and direct anatomical studies were evaluated with an AQUA-adapted framework addressing specimen selection, anatomical methods, outcome definition, reporting, and applicability [40]. Imaging and procedural studies were evaluated with the JBI analytical cross-sectional checklist, reconstructive clinical series with the JBI case-series checklist, and individual case reports with the JBI case-report checklist. The first appraisal was completed by J.J.C.-S. and independently verified by A.B.-M.; disagreements were resolved by consensus, with J.S.-G. available for adjudication. Ratings were categorized as low concern or some concern and informed interpretation and sensitivity analyses rather than automatic exclusion. Study-level judgments are reported in Supplementary Table S6.

### 2.7. Statistical Analysis

Analyses were performed in R version 4.5.2 using Metafor version 5.0-1 [41,42]. Proportions were transformed using the logit transformation; boundary proportions received the prespecified 0.5 continuity correction applied only to zero- or complete-event studies. Random-effects models used restricted maximum likelihood (REML) to estimate τ^2^ and Knapp–Hartung adjustment for 95% confidence intervals. Heterogeneity was summarized using I^2^ and τ^2^. Continuous variables were synthesized as pooled means when studies reported compatible units but were treated as exploratory when measurement landmarks or methods differed. For interpretive purposes, continuous outcomes with I^2^ > 75% were designated exploratory; their pooled means were reported only as descriptive summaries and were not used to define normative values or procedural thresholds. Meta-analysis required at least three compatible studies, and cadaveric prevalence was never pooled with in vivo visualization or procedural identification.

### 2.8. Management of Overlapping Reports

Potentially overlapping reports were evaluated before synthesis. Reports from the same cohort or institution were retained only when they contributed distinct outcome domains, and a cohort was not counted twice within the same pooled outcome. The Callese/Cusumano reports were separated by imaging outcome; Weitgasser/Kohlhauser overlap was controlled by variable; Iorio 2011/2012 were used for distinct anatomical domains; and duplicate bibliographic records were counted once.

### 2.9. Unit-of-Analysis Audit and Sensitivity Analysis

For each cadaveric study, the reported number of cadavers and anatomical units (knees, limbs, or thighs) was extracted. Because bilateral anatomical units from one donor may be correlated, the original unit-level model was supplemented by a conservative design-effect analysis. Sampling variances were multiplied by DE = 1 + (m − 1)ρ, where m was the reported mean number of anatomical units per cadaver and ρ was set to 1 as a worst-case within-cadaver correlation. When the cadaver count was unavailable, m = 2 was assumed. A second sensitivity analysis retained only studies with known cadaver counts. These analyses evaluate precision under clustering; they do not change observed study-level proportions. Study-level assumptions and reproducible code are provided in Supplementary Tables S7 and S8 and Supplementary File S1.

### 2.10. Strict and Broad Cadaveric Presence Datasets

The strict DGA-presence dataset was defined a priori to include studies with an unconstrained denominator in which DGA presence or absence could be observed across the sampled anatomical units. The broad dataset additionally included five studies in which DGA identification was structurally constrained by landmark, flap, angiosome, or perfusion-based designs: Başarir 2006 [43], Thiengwittayaporn 2009 [44], Iorio 2011 [13], Iorio 2012 [14], and Rahmanian-Schwarz 2011 [25]. The broad analysis was therefore retained as a sensitivity analysis rather than the primary estimate. Study-level inclusion decisions and rationales are reported in Supplementary Table S9.

## 3. Results

### 3.1. Study Selection

The database search identified 2186 records. After removal of 945 duplicates, 1241 records underwent title/abstract screening. Seventy-five reports were sought for retrieval, five were not retrieved, and 70 full texts were assessed. Sixty-five primary studies were retained for evidence synthesis; four context/citation-only records were not counted as primary evidence, and one non-human study was excluded. Figure 2 and Table 1 summarize the study-selection pathway. The targeted update performed on 18 August 2026 identified six post-cutoff publications [11,12,30,31,32,38]. They were retained for contextual discussion only and did not alter the original PRISMA counts, the 65-study evidence set, or the frozen quantitative datasets (Supplementary Table S10).

### 3.2. Study Characteristics and Evidence Strata

The 65 primary studies comprised 30 predominantly anatomical or physiological investigations (22 DGA-core and eight complementary genicular-network studies) and 35 clinical, pathological, or procedural investigations (eight imaging/procedural studies, five safety reports, and 22 reconstructive studies). Several reports contained mixed components; classification was based on their principal contribution to synthesis. All 65 primary studies were included in the qualitative synthesis and evidence map. Quantitative pooling was outcome-specific: 22 studies contributed to at least one meta-analysis, whereas the remaining 43 were retained in the narrative or evidence-map synthesis because they reported non-compatible outcomes or units, focused on complementary genicular vessels, or comprised imaging, reconstructive, or safety evidence without a compatible numerator and denominator. Thus, these studies were not omitted from the main synthesis; they were excluded only from statistical pooling. The overall quantitative synthesis hierarchy is summarized in Table 2, while the study-level characteristics and synthesis roles of all 65 primary studies are presented in Table 3 and Supplementary Table S5. The relationships among the principal evidence domains and their clinical translation are summarized in Figure 3.

### 3.3. Cadaveric DGA-Core Anatomy

The primary strict cadaveric analysis included 16 studies and yielded a pooled DGA presence of 91.6% (95% CI 86.6–94.8; I^2^ = 30.3%; Figure 4). The broad analysis included 21 studies and yielded 93.3% (89.6–95.8; I^2^ = 37.3%; Figure S1). The five broad-only studies were Başarir 2006 [43] and Thiengwittayaporn 2009 [44] (landmark/safe-zone studies with structurally constrained identification), Iorio 2011 [13] and Iorio 2012 [14] (flap/perfusion or angiosome designs), and Rahmanian-Schwarz 2011 [25] (reconstructive/flap preselection). Their inclusion increased the estimate only modestly, supporting retention of the strict analysis as the primary result.

### 3.4. DGA Origin and Branching Pattern

The DGA arose from the femoral/superficial femoral arterial system in 92.4% of observations (95% CI 89.2–94.7; I^2^ = 12.8%; Figure 5). Popliteal origin was uncommon at 3.2% (2.2–4.6; I^2^ = 0%; Figure S2). An articular/osteoarticular branch arose from the DGA in 90.3% (84.1–94.2; I^2^ = 35.7%; Figure 6). The saphenous branch/artery estimate was 79.5% (68.0–87.6; I^2^ = 71.8%) and remained supplementary because definitions varied (Figure S3). The pooled DGA diameter was 2.07 mm (1.79–2.36), with study means ranging from 1.62 to 2.70 mm and very high heterogeneity (I^2^ = 95.2%; Figure S4). The pooled origin distance from the joint line/interline was 13.35 cm (12.82–13.88; I^2^ = 80.2%; Figure S5). Because heterogeneity was very high for both continuous outcomes, these pooled means have limited clinical interpretability; they are reported only as exploratory summaries and should not be used as normative values or procedural cutoffs. Major reported variant configurations are summarized schematically in Figure S7.

### 3.5. In Vivo Imaging and Procedural Evidence

Three compatible GAE/imaging datasets contributed 637 examinations or procedures, with DGA visualization or identification in 612 (39/39, 197/205, and 376/393) [5,6,8]. The pooled visualization estimate was 95.9% (95% CI 93.1–97.6; I^2^ = 0%; Figure 7). This result represents procedural visualization rather than anatomical prevalence. CBCT and DSA studies demonstrated the value of three-dimensional vessel mapping for target selection and recognition of diminutive or overlapping vessels [6,7], whereas DSA studies characterized angiographic variants, branching patterns, and anastomotic networks relevant to GAE planning [5,8]. CTA and Doppler studies supported associations between DGA caliber or flow and osteoarthritic joint changes [9,10].

### 3.6. Reconstructive and Recipient-Vessel Evidence

Reconstructive studies described medial femoral condyle flaps, DGA perforator and chimeric flaps, saphenous-axis designs, and DGA or related genicular arteries as recipient vessels [13,14,15,16,17,18,19,20,21,22,23,24,25,26,27,28,29,55,56,57,58,59,60,61,62,63,64,65,66,67,68,69,72,73,74,75]. The evidence demonstrates anatomical feasibility and a range of reconstructive applications, but most clinical reports were small series or case reports and did not support pooled estimates of clinical effectiveness or safety.

### 3.7. Safety-Relevant Anatomy

Five primary safety reports described pseudoaneurysm, bleeding, or other DGA-branch pathology after arthroscopy, anterior cruciate ligament reconstruction, transient patellar dislocation, post-arthroplasty intervention, or rare branch-level disease [33,34,35,36,37]. Diagnosis was generally established using CTA, Doppler ultrasound, or angiography, and management included selective embolization or surgical treatment. Because this evidence was case-based, it was used to identify plausible vulnerable branches and diagnostic pathways rather than to estimate complication rates.

### 3.8. Quality Appraisal

Among the 65 primary studies, 26 were judged to have low methodological concern and 39 to have some concern; none was classified as high concern under the prespecified design-specific frameworks. Common concerns included incomplete reporting of donor characteristics, mixed anatomical units, nonstandard landmarks, retrospective or selected imaging cohorts, small reconstructive series, and limited reporting in historical case reports. Study-level AQUA/JBI judgments are provided in Supplementary Table S6.

### 3.9. Unit-of-Analysis Sensitivity

The unit audit identified studies reporting one or two anatomical units per cadaver and several reports in which the number of donors was unavailable. Under the deliberately conservative worst-case adjustment (ρ = 1; unknown cluster size m = 2), strict DGA presence was 89.7% (95% CI 84.7–93.2%), compared with 91.6% (86.6–94.8%) in the original unit-level model. Restricting the analysis to ten studies with known cadaver counts yielded 88.8% (80.9–93.6%). Cluster-adjusted estimates for femoral/SFA origin, popliteal origin, and branch outcomes were similarly close to the original estimates (Supplementary Table S8 and Figure S6). The substantive conclusions were therefore unchanged, although the sensitivity analysis provides a more conservative characterization of precision. The evidence domains and their principal clinical translation are summarized in Table 4.

## 4. Discussion

This systematic review provides a stratified synthesis of the clinical anatomy of the DGA and its genicular network. The main cadaveric finding was a strict pooled presence of 91.6%. Importantly, the estimate remained high after a worst-case adjustment for non-independent bilateral specimens (89.7%), indicating that the central conclusion was not driven by treating paired limbs as fully independent. The DGA is therefore usually identifiable, but absence, reduced caliber, alternative dominance, and uncommon popliteal origin remain relevant to planning.

The strict and broad analyses address different sampling constructs. The strict dataset estimates presence in samples where both presence and absence could be observed without structural preselection. The broad dataset added five landmark, flap, angiosome, or perfusion studies in which identification of the DGA was partly required by design. The small difference between estimates (91.6% versus 93.3%) is reassuring, but the strict estimate remains the less biased primary result.

Cadaveric anatomy and in vivo procedural visualization were complementary rather than interchangeable. Across 637 compatible GAE-related examinations or procedures, the DGA was identified in 612, yielding a pooled visualization estimate of 95.9% (Figure 7). This high value reflects imaging technique, contrast delivery, disease-related vascularity, field of view, and operator interpretation; it should not be interpreted as anatomical prevalence. Recent anatomical and technical reviews reach a similar conclusion: angiographic planning must integrate the variable collateral network rather than rely on a single textbook pattern [11,12].

The DGA may be relevant to GAE because its articular/osteoarticular branches can supply medial capsular and periarticular territories. Anastomoses with the SMGA and other genicular arteries make selective mapping important near superficial branches and collateral pathways. The present review did not evaluate clinical efficacy, and the available vessel-level studies do not justify prescriptive treatment thresholds. Rather, the synthesis supports anatomical plausibility and reinforces the value of CBCT/DSA mapping.

Reconstructive evidence demonstrates diverse uses of the DGA, saphenous branch, and related genicular vessels for medial femoral condyle, perforator, chimeric, and recipient-vessel strategies. Recent reconstructive and access reports extend this translational spectrum [30,32]. Nevertheless, the clinical evidence was dominated by small series and case reports. These publications support feasibility and generate procedural hypotheses but cannot establish comparative effectiveness, complication rates, or universal flap-selection rules.

The safety literature was similarly descriptive. Case reports of pseudoaneurysm, hemorrhage, and branch-level pathology show that genicular vascular injury can follow common periarticular procedures or mimic other lesions [33,34,35,36,37,38]. These observations identify plausible diagnostic and therapeutic pathways, but their rarity and publication bias preclude estimation of incidence. Accordingly, the clinical conclusions have been deliberately softened.

The pooled DGA diameter of 2.07 mm should be interpreted cautiously. Study-level means ranged from 1.62 to 2.70 mm, and heterogeneity was very high because preservation method, measurement site, vessel definition, and internal versus external diameter differed. Origin-distance estimates were likewise highly heterogeneous because anatomical landmarks and measurement methods varied. Both continuous outcomes were therefore retained only as exploratory summaries. Their pooled means have limited clinical significance, no normative range or procedural cutoff is proposed, and study-specific estimates should be prioritized over the pooled values.

The principal strengths of this review are its prespecified protocol, broad database coverage, explicit evidence stratification, complete bibliographic traceability of 65 primary studies, design-specific appraisal, and outcome-specific quantitative synthesis. Limitations include heterogeneous terminology, incomplete donor-level reporting, mixed units of analysis, potential cohort overlap, and the small size of many reconstructive and safety studies. The worst-case unit-of-analysis sensitivity reduced concern about paired limbs but could not replace individual-participant or donor-cluster data. Formal reporting-bias tests and GRADE were not applied because most outcomes were anatomical, heterogeneous, or supported by fewer than ten studies.

Future anatomical studies should report both cadaver and limb denominators, bilateral pairing, preservation method, measurement landmarks, and standardized branch nomenclature. Imaging studies should provide complete vessel-level denominators and acquisition parameters. Prospective clinical cohorts are required to determine whether specific anatomical variants affect GAE targeting, reconstructive outcomes, or safety.

## 5. Conclusions

The DGA is usually present and most often arises from the femoral/superficial femoral arterial system, but clinically relevant variation exists in origin, dominance, caliber, and branch configuration. Conservative adjustment for bilateral cadaveric sampling did not materially change the primary presence estimate. Cadaveric anatomy and procedural imaging should be interpreted as complementary evidence domains. These anatomical findings may inform GAE mapping and reconstructive planning; however, exploratory morphometric pooled means should not be interpreted as clinical thresholds, and reconstructive and safety conclusions require confirmation in larger prospective cohorts.

## Figures and Tables

**Figure 1:**
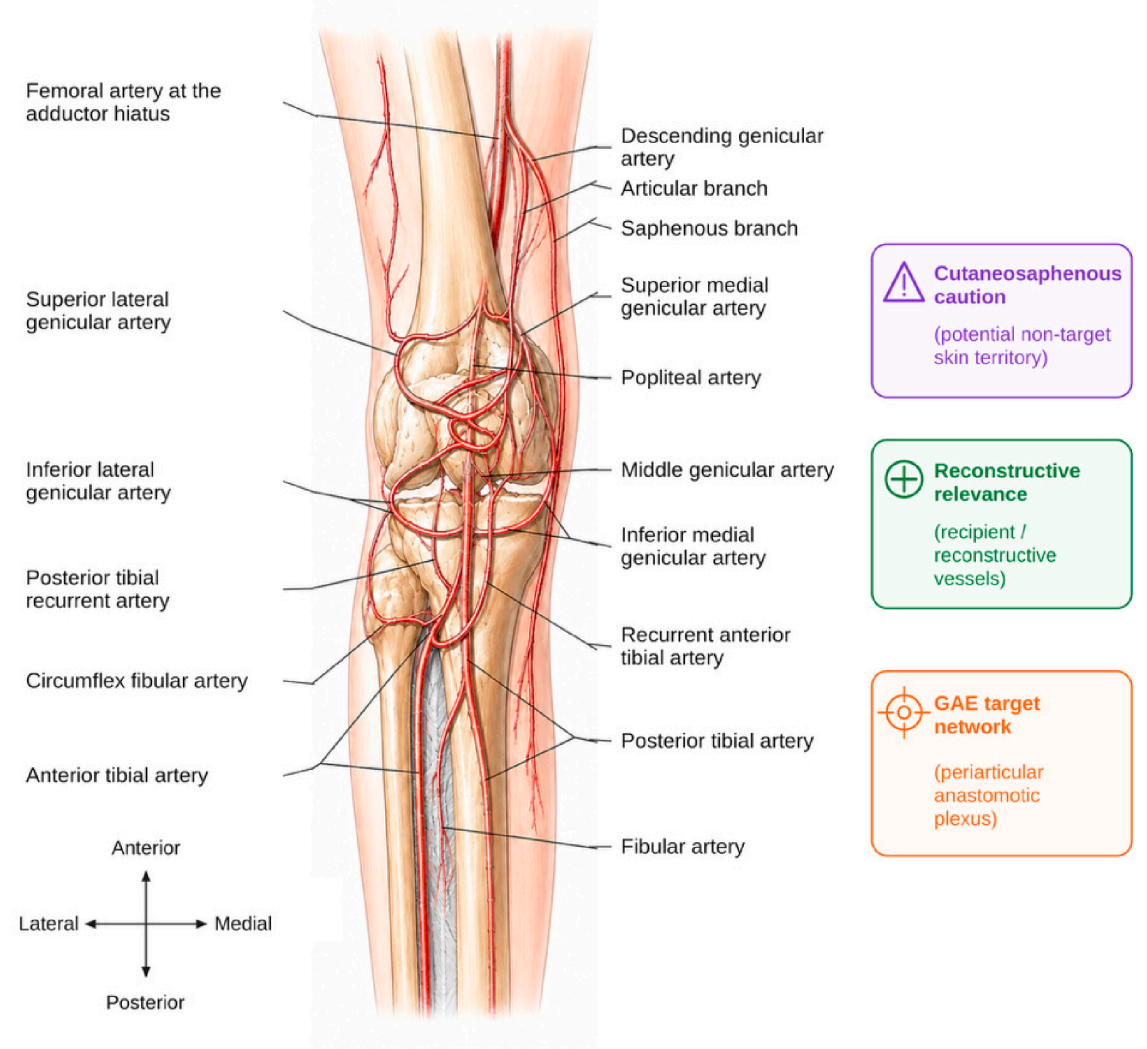
Clinical anatomical map of the descending genicular artery and genicular arterial network around the knee. The initial visual draft was generated using OpenAI ChatGPT Images 2.0 with the GPT Image model available in the ChatGPT web interface (OpenAI, San Francisco, CA, USA; accessed 7–17 July 2026). The authors subsequently edited and relabeled the image, standardized vessel nomenclature to current English anatomical usage—including the superior and inferior medial and lateral genicular arteries, middle genicular artery, circumflex fibular artery, fibular artery, and recurrent anterior tibial artery—and anatomically validated the final figure. The authors take full responsibility for its accuracy and final content. The map highlights the DGA and its major branches, complementary genicular vessels, GAE target territories, reconstructive relevance, and cutaneosaphenous non-target caution.

**Figure 2:**
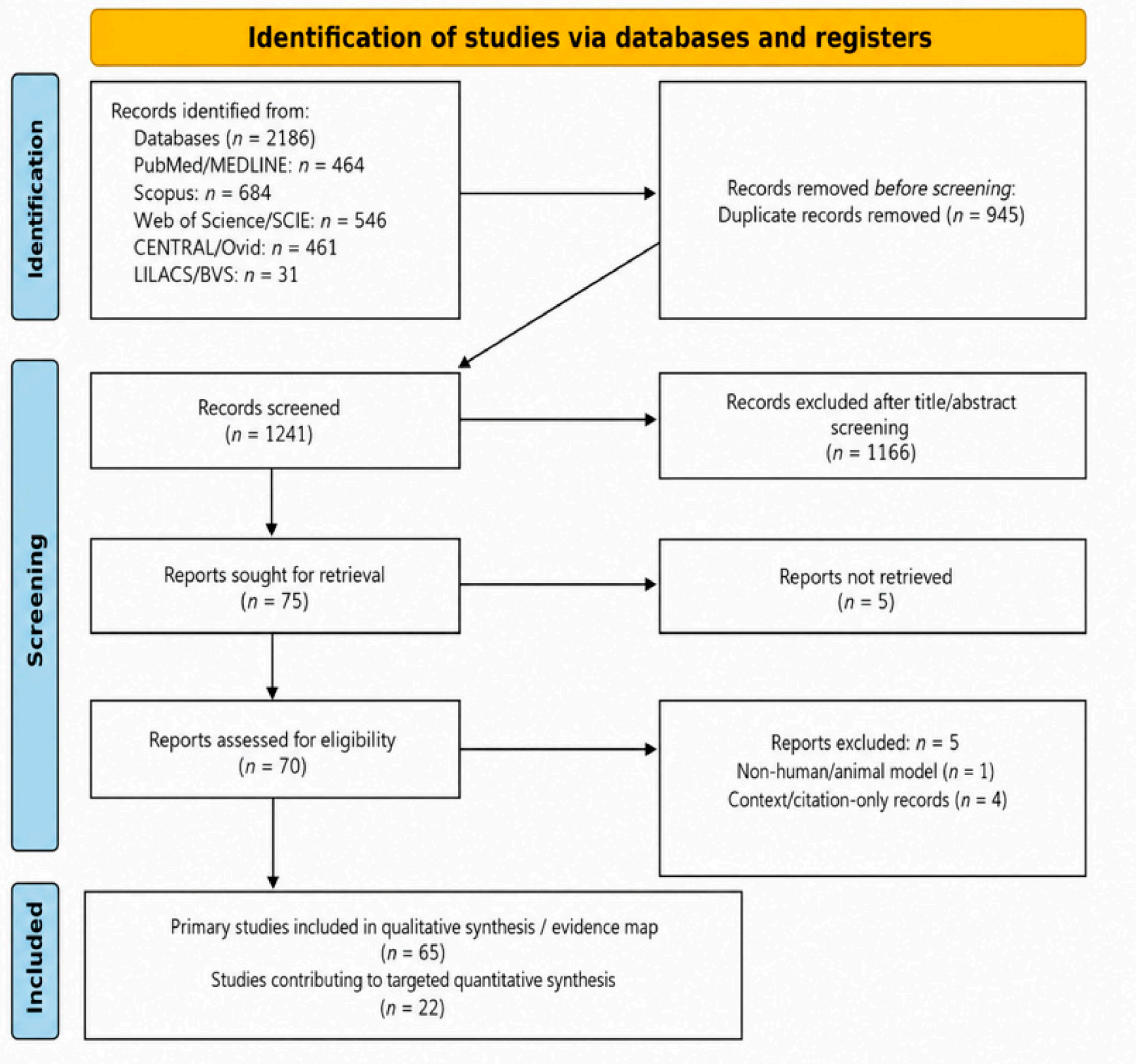
PRISMA 2020 flow diagram summarizing identification, screening, eligibility, and inclusion. Only the declared database-search pathway is shown. Citation checking was performed for contextual verification and did not add additional records to the PRISMA flow. The quantitative synthesis count refers to studies contributing to at least one targeted meta-analytic outcome; not all studies contributed to every pooled estimate.

**Figure 3:**
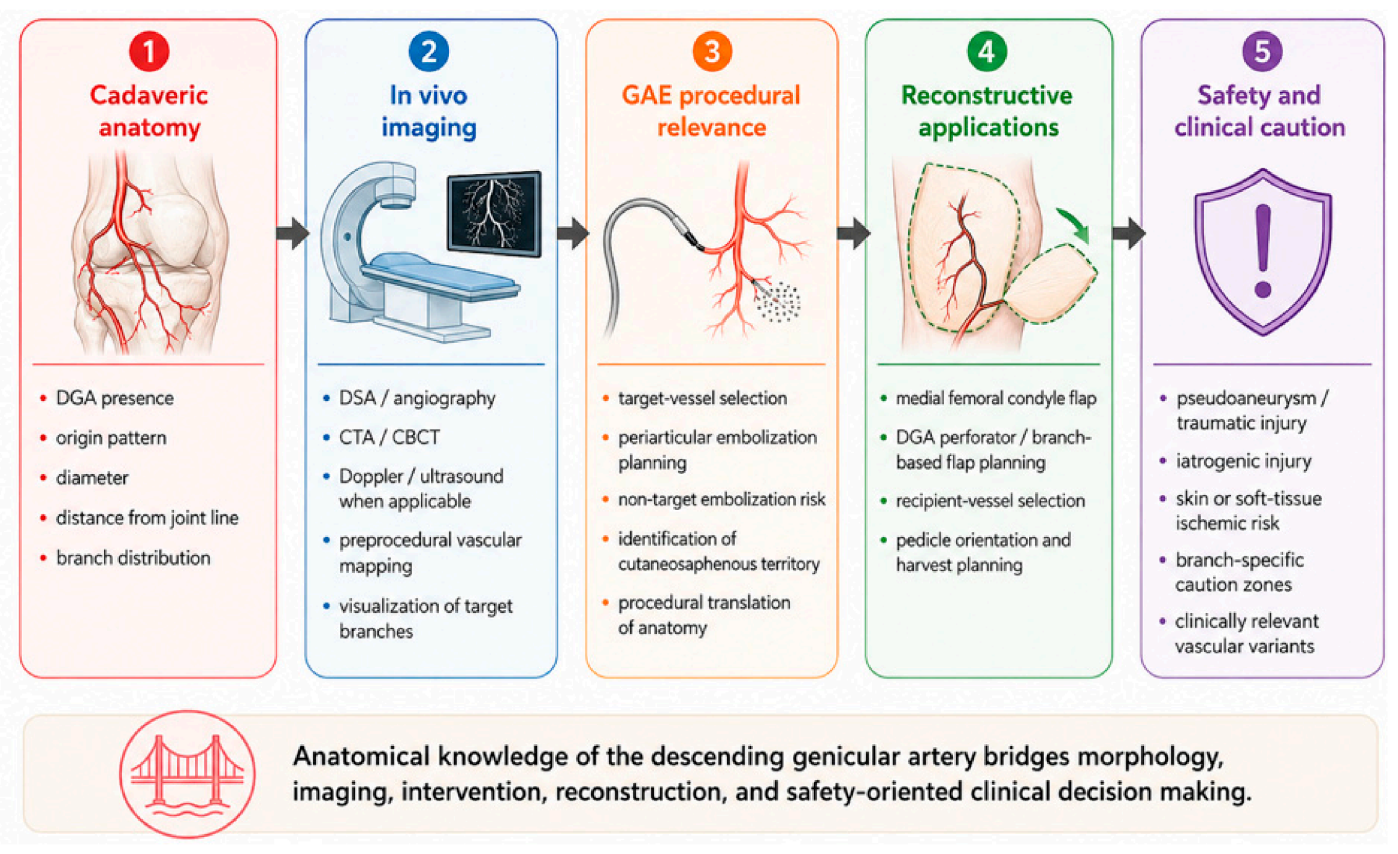
Translational evidence map linking cadaveric anatomy, in vivo imaging, GAE, reconstructive applications, and safety-relevant reports. The initial visual draft was generated using OpenAI ChatGPT Images 2.0 with the GPT Image model available in the ChatGPT web interface (OpenAI, San Francisco, CA, USA; accessed on 7–17 July 2026). The authors manually revised and scientifically validated all concepts, labels, and relationships and take full responsibility for the accuracy and final content. A reformatted version is supplied as the graphical abstract.

**Figure 4:**
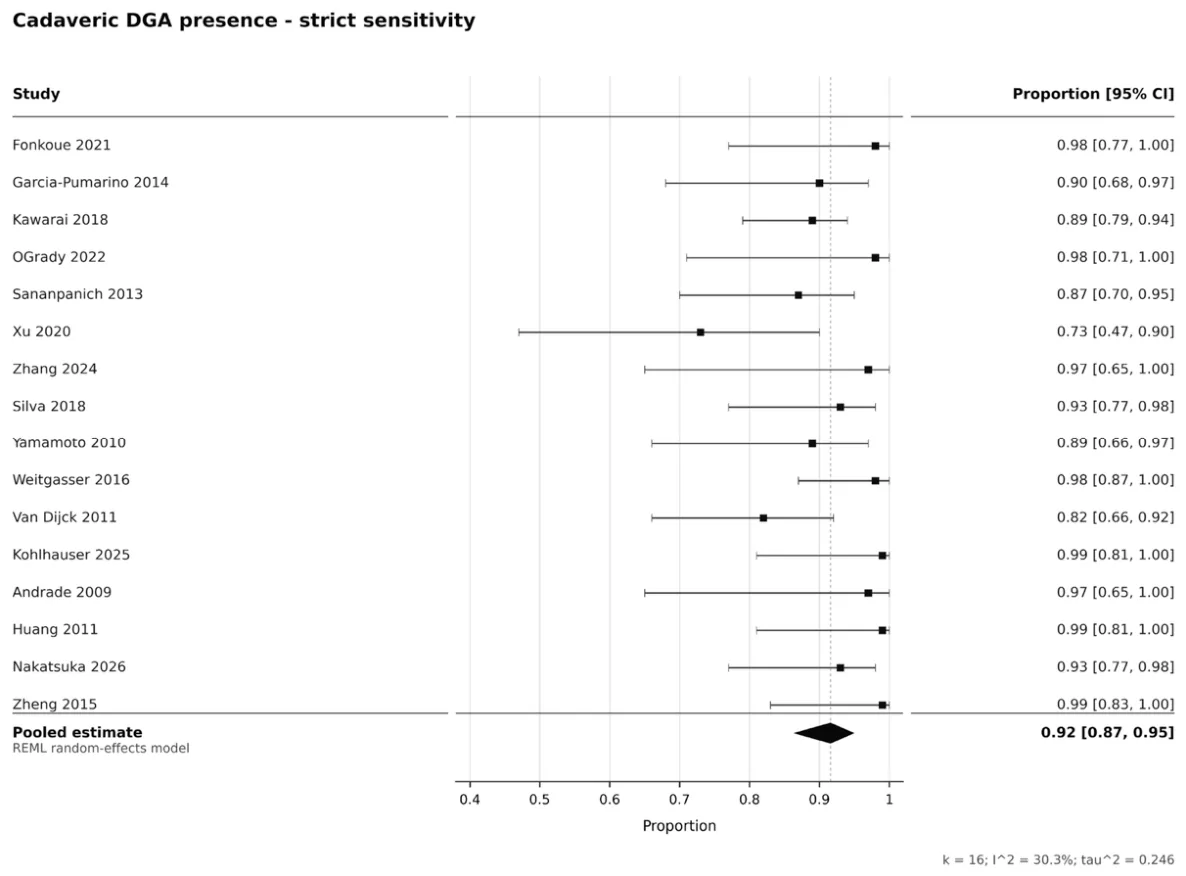
Forest plot of DGA presence in the strict cadaveric sensitivity dataset. The estimate is restricted to studies with compatible cadaveric denominators and excludes analyses in which DGA identification was structurally preselected or landmark-dependent [1,2,3,4,15,16,17,19,20,21,22,24,26,45,46,47].

**Figure 5:**
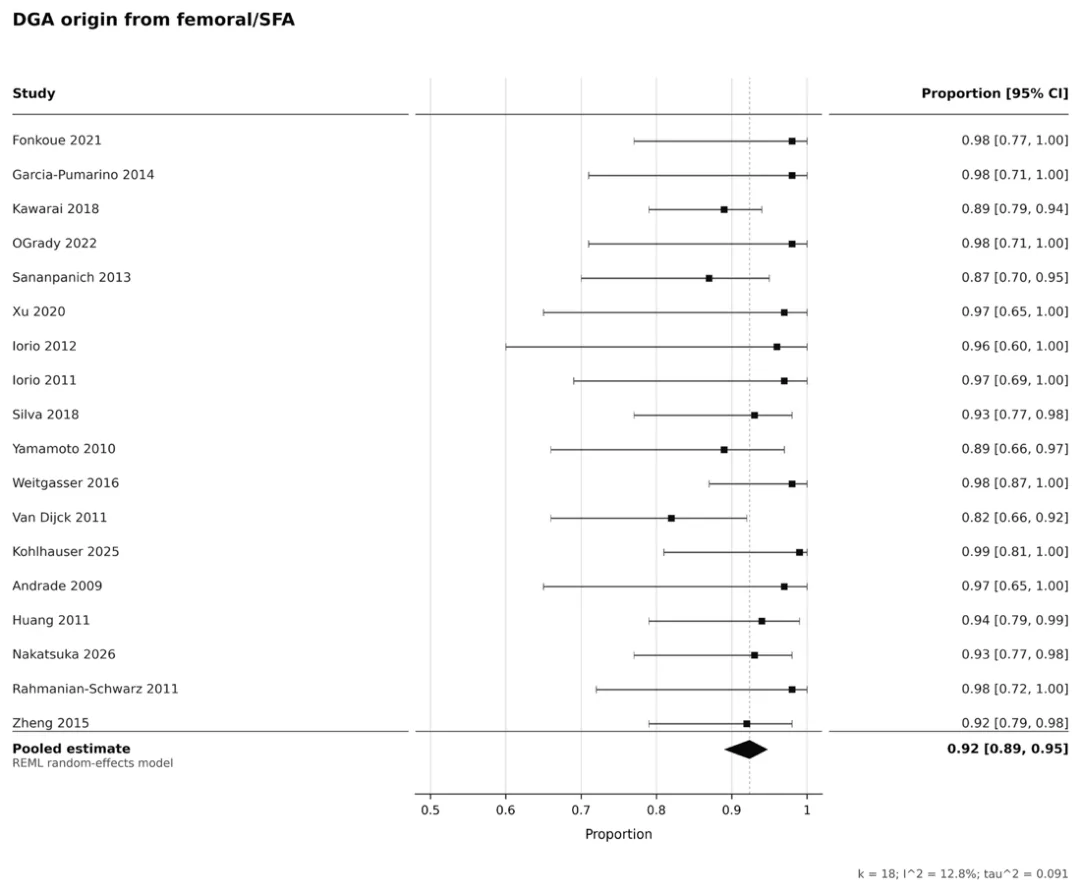
Forest plot of DGA origin from the femoral or superficial femoral arterial system in cadaveric studies. This synthesis was treated as a secondary anatomical outcome [1,2,3,4,13,14,15,16,17,19,20,21,24,25,26,45,46,47].

**Figure 6:**
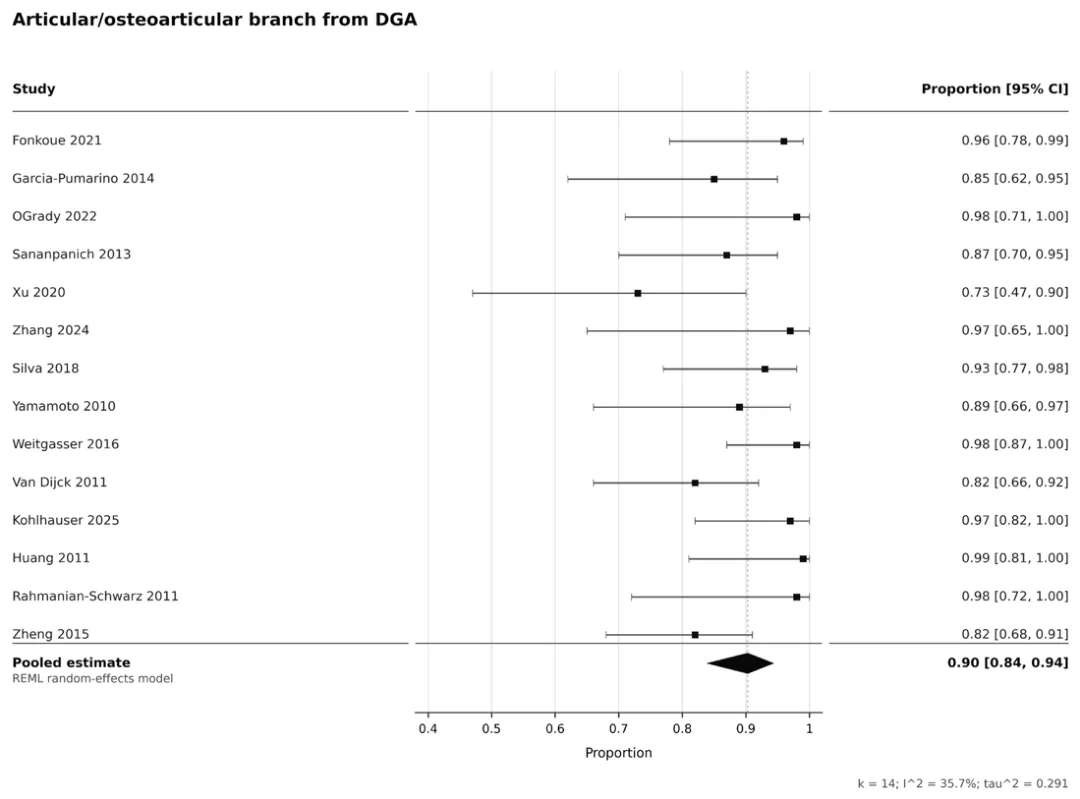
Forest plot of articular or osteoarticular branch origin from the DGA in cadaveric studies. Original anatomical nomenclature was harmonized only when the branch supplied the medial femoral condyle, periosteum, articular capsule, or osteoarticular territory [1,2,3,4,15,16,17,19,21,22,25,26,45,47].

**Figure 7:**
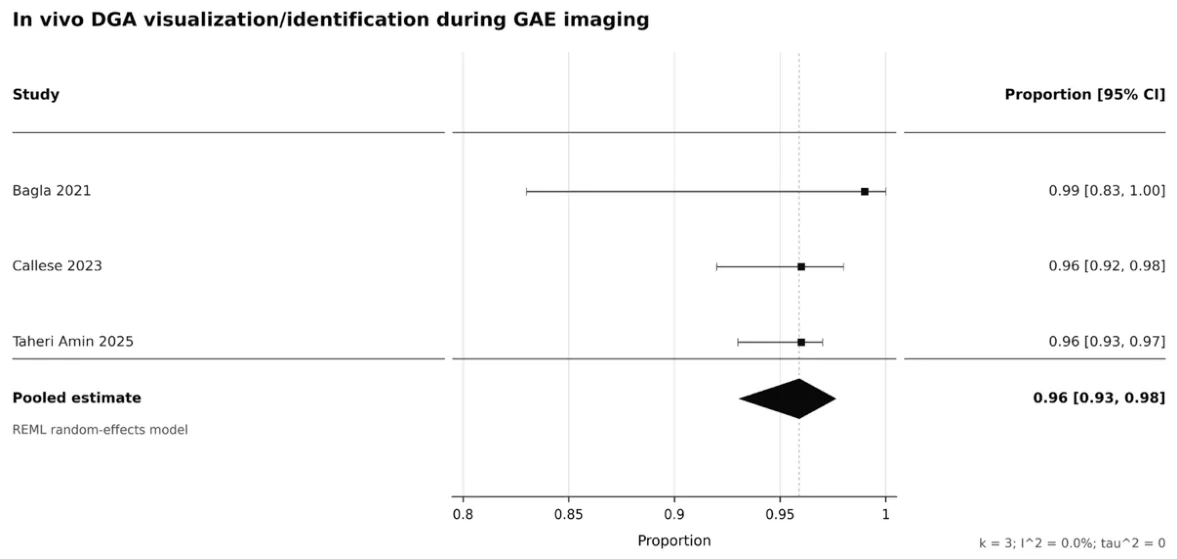
Forest plot of in vivo DGA visualization or identification during GAE-related imaging. These estimates reflect procedural visualization/identification and were not pooled with cadaveric anatomical prevalence estimates [5,6,8].

**Table 1:** PRISMA 2020 flow and evidence-stratification summary.

Item	n	Interpretation
Records identified from databases	2186	Database identification step.
Duplicates removed before screening	945	Deduplicated in Zotero with manual verification.
Records screened by title/abstract	1241	Screening denominator.
Reports sought for retrieval	75	Potentially eligible reports after title/abstract screening.
Reports not retrieved	5	Unavailable after final retrieval attempts.
Full-text reports assessed	70	Reports assessed for eligibility.
Primary studies retained for synthesis	65	Primary evidence across strata A–D.
Context/citation-only records	4	Not counted as primary evidence.
Non-human study excluded	1	Excluded from primary synthesis.

**Table 2:** Final quantitative synthesis hierarchy.

Outcome	k	Estimate (95% CI)	Heterogeneity	Reporting Destination
DGA presence, strict cadaveric sensitivity	16	91.6% (86.6–94.8)	I^2^ = 30.3%	Primary result; main forest plot.
DGA presence, broad cadaveric extraction	21	93.3% (89.6–95.8)	I^2^ = 37.3%	Sensitivity/supplementary.
DGA origin from femoral/SFA system	18	92.4% (89.2–94.7)	I^2^ = 12.8%	Secondary result.
DGA origin from popliteal artery	17	3.2% (2.2–4.6)	I^2^ = 0%	Rare variant; secondary result.
Articular/osteoarticular branch from DGA	14	90.3% (84.1–94.2)	I^2^ = 35.7%	Secondary result.
Saphenous branch/artery from DGA	15	79.5% (68.0–87.6)	I^2^ = 71.8%	Exploratory; supplementary.
DGA diameter and origin distance	9/9	2.07 mm/13.35 cm	I^2^ = 95.2%/80.2%	Exploratory only; high heterogeneity precludes normative or clinical-threshold interpretation.
DGA visualization during GAE imaging	3	95.9% (93.1–97.6)	I^2^ = 0%	Separate in vivo imaging result.
DGA presence, worst-case cluster-adjusted sensitivity	16	89.7% (84.7–93.2)	I^2^ ≈ 0%	Reviewer-driven unit-of-analysis sensitivity.
DGA presence, known cadaver count only	10	88.8% (80.9–93.6)	I^2^ = 1.9%	Sensitivity restricted to known donor counts.

**Table 3:** Characteristics and synthesis roles of the 65 primary studies retained for qualitative evidence synthesis and evidence mapping.

Study	Design/Modality	Evidence Stratum	Principal Contribution	Synthesis Role
Fonkoue 2021 [1]	Cadaveric dissection	Cadaveric DGA-core	DGA presence; origin/distance; branching; branches; image-guided implications	Contributed to cadaveric DGA quantitative synthesis
García-Pumarino 2014 [2]	Cadaveric dissection	Cadaveric DGA-core	Branching pattern; diameter; origin distance; articular branch	Contributed to cadaveric DGA quantitative synthesis
Yamamoto 2010 [3]	Fresh-frozen cadaveric dissection/injection	Cadaveric MFC/DGA anatomy	DGA presence; OAB; SA; SMGA; perforators	Contributed to cadaveric DGA/SMGA quantitative synthesis
Van Dijck 2011 [4]	Cadaveric dissection	Cadaveric MFC/DGA anatomy	DGA dominance; DGA absence; length; origin; diameter; SMGA	Contributed to cadaveric dominance/presence synthesis
Bagla 2021 [5]	DSA during GAE	Angiographic/procedural GAE anatomy	DGA presence; M1/M2/L1/L2; targets; anastomoses	Contributed to angiographic DGA/target/anastomosis synthesis
Callese 2023 [6]	Cone-beam CT during GAE	CBCT/GAE anatomical imaging	DGA visualization; CBCT variants; genicular branching	Contributed to imaging DGA visualization synthesis
Cusumano 2023 [7]	CBCT and DSA during GAE	CBCT + DSA procedural imaging	Targets identified; DGA target; CBCT vs. DSA	Modality comparison only
Taheri Amin 2025 [8]	Digital subtraction angiography	DSA/GAE anatomy	DGA identification; anastomoses; branching; vessel diameters	Contributed to angiographic quantitative synthesis
Behzadi 2025 [9]	CTA	CTA vascular physiology	DGA caliber; KL grade; DGA/SFA ratio	Narrative DGA caliber/osteoarthritis-severity synthesis
Fukumoto 2021 [10]	Ultrasound Doppler	Doppler ultrasound physiology	DGA peak systolic velocity; KOA; effusion; pain	Physiology only
Iorio 2011 [13]	Cadaveric angiographic perfusion	Cadaveric periosteal perfusion	DGA presence; origin distance; pedicle length; perfusion extent	Contributed to cadaveric morphometry/perfusion synthesis
Iorio 2012 [14]	Cadaveric injection + clinical series	Cadaveric angiosome + clinical	DGA-CB; SAB; angiosome area; clinical use	Contributed to cadaveric angiosome/perforator synthesis
Sananpanich 2013 [15]	Cadaveric dissection	Cadaveric DGA/reconstructive	DGA presence; SA; perforators; skin territory	Contributed to cadaveric DGA/perforator quantitative synthesis
Silva 2018 [16]	Cadaveric dissection	Cadaveric DGA/MFC anatomy	DGA presence; SA from DGA; distance; length; diameter	Contributed to cadaveric DGA quantitative synthesis
Weitgasser 2016 [17]	Thiel cadaveric dissection	Cadaveric MFC/DGA anatomy	DGA presence; Y/H pattern; SA; AB; diameter/length	Contributed to cadaveric DGA branching/saphenous-branch synthesis
Kim 2026 [18]	CTA + clinical/reconstructive	CTA anatomical applied DGA flap	DGA visualized on CTA; cutaneous supply; SA/perforators	Contributed to imaging DGA/saphenous/perforator synthesis
Tozzi 1996 [33]	CT + angiography	DGA pseudoaneurysm case report	DGA pseudoaneurysm; arthroscopy; surgical resection	Narrative synthesis; not pooled
Tsubosaka 2017 [34]	CT + Doppler + embolization	DGA articular branch pseudoaneurysm	Articular branch injury; ACL reconstruction; embolization	Narrative synthesis; not pooled
Silvestri 2018 [35]	CT angiography + embolization	DGA injury case report	DGA injury; embolization; trauma	Narrative synthesis; not pooled
Oláh 2025 [36]	Angiography/embolization case report	GAE case report post-TKA pain	DGA embolization; post-TKA pain; PVA; WOMAC/VAS narrative	Narrative synthesis; not pooled
O’Grady 2022 [19]	Cadaveric dissection + angiography	Cadaveric + angiographic correlation	DGA branching; DGA-SMGA anastomosis; translational anatomy	Use cadaveric and angiographic components separately
Kawarai 2018 [20]	Cadaveric dissection	Cadaveric DGA-core	DGA presence; classification; origin distance	Contributed to cadaveric DGA quantitative synthesis
Kohlhauser 2025 [21]	Thiel cadaveric dissection	Cadaveric combined flap anatomy	DGA detectable; AB/SA/MB branching; combined flaps	Contributed to cadaveric DGA branching/saphenous-branch synthesis
Zhang 2024 [22]	Cadaveric dissection + clinical series	Cadaveric + clinical reconstructive	DGAP-SV distance; flap outcomes; chimeric flaps	Narrative synthesis; comparable DGAP–saphenous-vein variables only
Mohan 2019 [23]	Cadaveric CTA + clinical series	Perforasome/reconstructive anatomy	Perforators from SFA/SA/DGA; perforasome area; clinical cases	Perforator origin/perforasome subgroup only
Nakatsuka 2026 [24]	Cadaveric dissection + clinical application	Cadaveric + clinical DGA recipient anatomy	DGA presence; vastus medialis branch presence/caliber	Contributed to branch-specific cadaveric synthesis
Rahmanian-Schwarz 2011 [25]	Cadaveric dissection + clinical cases	Cadaveric + clinical MFC/DGA anatomy	DGA presence; diameter; origin; trifurcation; AB/MB/SB	Included once in cadaveric DGA synthesis
Zheng 2015 [26]	Cadaveric dissection + clinical cases	Cadaveric + clinical DGA perforator	OAB perforator; caliber; location; propeller flap	Contributed to DGA perforator subgroup synthesis
Kobayashi 2022 [27]	Case series	DGA recipient vessel case series	DGA recipient vessel for free flaps	Narrative synthesis; not pooled
Li 2023 [28]	Clinical series	DGA recipient vessel clinical series	DGA/saphenous/articular branch recipient vessels	Narrative synthesis; descriptive counts only
Venkatramani 2014 [29]	Clinical series	DGA recipient vessel clinical series	DGA/muscular branches recipient vessels	Narrative synthesis; not pooled
Xu 2020 [45]	Cadaveric dissection	Cadaveric DGA/reconstructive	DGA presence; SA; osteoarticular branch; chimeric flaps	Contributed to cadaveric DGA/perforator quantitative synthesis
Başarir 2006 [43]	Cadaveric dissection	Cadaveric surgical landmark anatomy	DGA entry angle; safe distance from patella	Landmark/morphometry only
Thiengwittayaporn 2009 [44]	Cadaveric dissection	Cadaveric surgical landmark anatomy	DGA safe zone; distance to patella; entry angle	Landmark/morphometry only
Andrade 2009 [46]	Cadaveric dissection	Cadaveric MFC/DGA anatomy	DGA distance; diameter; pedicle length; fasciocutaneous branch	Contributed to cadaveric DGA quantitative synthesis
Huang 2011 [47]	Cadaveric dissection + clinical series	Cadaveric + clinical DGA/adductor tendon	DGA origin/diameter; branches; adductor tendon flap	Contributed to cadaveric DGA quantitative synthesis
Sighary/Sajan 2022 [48]	Cadaveric dissection	Cadaveric genicular anatomy	Genicular branching; DGA types	DGA branching only; not DGA presence
Tinhofer 2018 [49]	HREM microanatomy	Dermal microvascular DGA angiosome	Dermal arterial units; overlap anastomoses	Narrative microanatomical synthesis; not pooled
Romiti 2006 [50]	Arteriography	Arteriographic collateral anatomy	DGA presence/dominance; perigeniculate collateral network	Pathologic angiographic subgroup only
Hayashi 1990 [51]	Cadaveric dissection + clinical cases	SMGA/reconstructive anatomy	SMGA origin; flap anatomy	Non-DGA genicular subgroup only
Hayashi 1990 [52]	Cadaveric dissection + clinical cases	SLGA/reconstructive anatomy	SLGA origin; lateral genicular flap anatomy	Non-DGA genicular subgroup only
Blagojević 2015 [53]	Cadaveric dissection	Middle genicular artery anatomy	MGA origin/course/diameter	Non-DGA MGA subgroup only
Hirtler 2016 [54]	Cadaveric dissection	IMGA/pes anserinus anatomy	IMGA vascularization; anastomoses; ACL relevance	Non-DGA IMGA subgroup only
Hsieh 2023 [55]	Clinical series	DGAP flap clinical series	DGAP flap clinical outcomes	Narrative synthesis; not pooled
Christen 2022 [56]	Clinical series	MFC free flap clinical series	MFC flap clinical outcomes	Narrative synthesis; not pooled
Guzzini 2022 [57]	Clinical series + MRA subset	MFC donor-site safety	Donor-site morbidity; vascular regeneration	Narrative synthesis; not pooled
Guzzini 2016 [58]	Case report	Reconstructive case report	Pedicled MFC flap application	Narrative synthesis; not pooled
Grant 2005 [59]	Case report	Reconstructive case report	MFC vascularized graft application	Narrative synthesis; not pooled
Oh 2019 [60]	Clinical intraoperative series	Clinical intraoperative MFC anatomy	DGA presence/dominance; SMGA presence/dominance	Clinical intraoperative subgroup only
Pelzer 2010 [61]	Clinical series	MFC osteoperiosteal-cutaneous flap	DGA unnamed branch/SAB skin paddle	Narrative synthesis; not pooled
Mattos 2019 [62]	Clinical series	MFC flap clinical application	MFC flap clinical outcomes	Narrative synthesis; not pooled
Kumta 2017 [63]	Clinical series	MFC graft clinical application	MFC graft outcomes	Narrative synthesis; not pooled
Low 2022 [64]	Clinical/reconstructive	SLGA flap context	SLGA reconstructive context	Narrative synthesis; not pooled
Lyrtzis 2025 [37]	Case report	DGA branch pathology case report	DGA musculoarticular branch pathology	Narrative synthesis; not pooled
Park 2001 [65]	Clinical series	DGA/SMGA recipient vessels	DGA/SMGA recipient vessels; flap survival	Narrative synthesis; not pooled
Rees-Lee 2011 [66]	Clinical series	SMGA recipient vessel	SMGA recipient vessel	Narrative synthesis; not pooled
Shen 2012 [67]	Clinical series	Medial genicular network reconstruction	Ramus saphenous/DGA network; IMGA/PTA anastomoses	Narrative synthesis; not pooled
Shirota 2024 [68]	Case report	DGA recipient vessel case report	DGA recipient vessel; SCIP flap	Narrative synthesis; not pooled
Taniguchi 2009 [69]	Case report	SLGA flap clinical case	SLGA flap post-TKA	Narrative synthesis; not pooled
Salaria 2008 [70]	Cadaveric dissection	Middle genicular artery anatomy	MGA course/diameter/length	Non-DGA MGA subgroup only
Shahid 2015 [71]	Cadaveric dissection	Genicular arteries/MGA anatomy	SMGA/SLGA/IMGA/ILGA/MGA variations	Non-DGA genicular subgroup only
Zhang 2018 [72]	Clinical series	DGA-SA perforator flap clinical series	DGA-SA perforator flap outcomes	Narrative synthesis; not pooled
Xu 2023 [73]	Cadaveric dissection + clinical cases	SLGA perforator anatomy/clinical	SLGA perforator anatomy; clinical reconstruction	Non-DGA SLGA subgroup only
Yang 2021 [74]	Clinical series	SLGA perforator clinical series	SLGA perforator flap outcomes	Narrative synthesis; not pooled
Yalçın 2025 [75]	Cadaveric dissection + clinical cases	IMGA osteocutaneous flap anatomy/clinical	IMGA-based osteocutaneous flap	Non-DGA IMGA subgroup only

**Note:** References [1,2,3,4,5,6,7,8,9,10,13,14,15,16,17,18,19,20,21,22,23,24,25,26,27,28,29,33,34,35,36,37,43,44,45,46,47,48,49,50,51,52,53,54,55,56,57,58,59,60,61,62,63,64,65,66,67,68,69,70,71,72,73,74,75] correspond to the 65 primary studies retained for qualitative evidence synthesis and evidence mapping. References [39,40,41,42] are methodological or reporting sources. References [11,12,30,31,32,38] are post-cutoff contextual publications and are not counted among the 65 primary studies.

**Table 4:** Evidence domains and clinical translation.

Evidence Domain	Representative Contribution	Clinical Relevance
Cadaveric DGA-core anatomy	Presence, origin, branching, saphenous and articular/osteoarticular branches.	Defines anatomical substrate and variation relevant to surgery and embolization.
In vivo imaging/procedure	CBCT, DSA, CTA, Doppler and GAE vessel-level evidence.	Supports pre-procedural mapping and target-vessel identification.
Reconstructive applications	MFC flap, DGAP flap, saphenous-axis flaps and recipient-vessel use.	Guides flap design, pedicle choice and recipient-vessel selection.
Safety-relevant reports	Pseudoaneurysm, iatrogenic or traumatic DGA branch injury.	Highlights vulnerable branches and diagnostic/therapeutic strategies.

## Data Availability

Aggregate extraction data, quality-appraisal judgments, unit-of-analysis audit, frozen quantitative datasets, and analysis code are supplied with the revised Supplementary Materials (Supplementary File S1). No individual participant data were generated.

## Supplementary Materials

The following supporting information can be downloaded at: https://www.mdpi.com/article/10.3390/diagnostics16213642/s1, Table S1: Database-specific search strategies and yields; Table S2: PRISMA 2020 checklist; Table S3: Reports not retrieved, full-text exclusions and context/citation-only records; Table S4: Quality-appraisal summary by evidence stratum; Table S5: Bibliographic reconciliation and study-level traceability for the 65 primary studies; Table S6: Study-level methodological quality appraisal of the 65 primary studies; Table S7: Cadaveric unit-of-analysis audit; Table S8: Unit-of-analysis sensitivity results; Table S9: Study-level definition of the strict and broad cadaveric DGA-presence datasets; Table S10: Targeted recent-literature update performed on 18 August 2026; Figure S1: Broad cadaveric DGA presence analysis. This sensitivity analysis includes studies in which DGA identification may have been partially constrained by study design [1,2,3,4,13,14,15,16,17,19,20,21,22,24,25,26,43,44,45,46,47]; Figure S2: Cadaveric synthesis of DGA origin from the popliteal artery. This rare-variant estimate was retained as supplementary secondary anatomical evidence [2,3,4,13,14,15,16,17,19,20,21,24,25,26,45,46,47]; Figure S3: Saphenous branch or saphenous artery arising from the DGA. This synthesis was retained as supplementary because terminology and anatomical definitions varied across studies [1,2,3,4,14,15,16,17,19,21,22,25,26,45,47]; Figure S4: DGA diameter in cadaveric studies. This continuous outcome was retained as exploratory because preservation method, measurement site, and internal versus external diameter reporting varied substantially [4,15,16,17,21,26,45,46,47]; Figure S5: Distance from DGA origin to the knee joint line or interline. This continuous outcome was retained as exploratory because anatomical landmarks and measurement methods were heterogeneous [1,4,13,14,15,16,20,21,46]; Figure S6: Unit-of-analysis sensitivity for strict cadaveric DGA presence. The worst-case design-effect analysis assumed perfect within-cadaver correlation (ρ = 1) and, when the donor count was unavailable, two anatomical units per cadaver [1,2,3,4,15,16,17,19,20,21,22,24,26,45,46,47]; Figure S7: Author-generated schematic of major reported DGA/genicular configurations: common DGA branching, popliteal origin, separate saphenous and articular/osteoarticular branches, an independently arising saphenous artery, and SMGA-dominant supply. The schematic summarizes reported patterns and is not intended to establish a universal classification [1,2,3,4,13,14,15,16,17,19,20,21,22,23,24,25,26,43,44,45,46,47,48,49,50,51,52,53,54]; File S1: Analysis code and frozen datasets archive, provided separately. The Supplementary Materials use the same definitive citation numbering as the main manuscript, and all references cited therein are included in the main reference list [1,2,3,4,5,6,7,8,9,10,11,12,13,14,15,16,17,18,19,20,21,22,23,24,25,26,27,28,29,30,31,32,33,34,35,36,37,38,39,40,41,42,43,44,45,46,47,48,49,50,51,52,53,54,55,56,57,58,59,60,61,62,63,64,65,66,67,68,69,70,71,72,73,74,75].

## Abbreviations

The following abbreviations are used in this manuscript:

Abbreviation	Definition
CBCT	Cone-beam computed tomography
CTA	Computed tomography angiography
DGA	Descending genicular artery
DSA	Digital subtraction angiography
GAE	Genicular artery embolization
IMGA	Inferior medial genicular artery
MFC	Medial femoral condyle
SA	Saphenous artery
SFA	Superficial femoral artery
SLGA	Superior lateral genicular artery
SMGA	Superior medial genicular artery
ILGA	Inferior lateral genicular artery
MGA	Middle genicular artery
RATA	Recurrent anterior tibial artery
AQUA	Anatomical Quality Assurance
REML	Restricted maximum likelihood
AB/OAB	Articular/osteoarticular branch
ACL	Anterior cruciate ligament
DGAP	Descending genicular artery perforator
JBI	Joanna Briggs Institute
