# Supplementary material for: Clinical Anatomy of the Descending Genicular Artery and Genicular Arterial Network for Embolization and Reconstructive Surgery: A Systematic Review and Evidence Map with Targeted Meta-Analysis
Source: Diagnostics (Basel). 2026 Nov 4;16(21):3642. doi: 10.3390/diagnostics16213642 (PMC13761717; doi:10.3390/diagnostics16213642)
Supplement: Supplementary file 1 [file diagnostics-16-03642-s001.zip › diagnostics-4490355-supplementary.pdf]

Supplementary Materials

Clinical Anatomy of the Descending Genicular Artery and Genicular Arterial Network for Embolization and Reconstructive Surgery: A Systematic Review and Evidence Map with Targeted Meta-Analysis

Bibliographic reconciliation statement. The Supplementary Materials use the same definitive citation numbering as the main manuscript, and every formal reference cited as supporting evidence in this document is included in the main reference list. Bibliographic records listed exclusively in Table S3 to document the PRISMA study-selection process—including reports not retrieved, full-text exclusions, and context/citation-only records—are presented solely for screening and eligibility traceability and are not treated as cited references or as evidence contributing to the synthesis. The 65 primary studies retained for qualitative evidence synthesis/evidence mapping are individually traceable in Table S5 and correspond to references [1–10,13–29,33–37,43–75] in the main manuscript. References [39–42] are methodological/reporting sources, whereas references [11,12,30–32,38] are post-cutoff contextual publications and are not counted among the 65 primary studies.

Figure-development note. Figures 1 and 3 in the main manuscript were developed by the authors with generative-AI assistance and were subsequently manually revised and scientifically validated. Generative AI was not used for eligibility decisions, data extraction, quality appraisal, statistical analysis, interpretation, or scientific conclusions.

Table S1. Database-specific search strategies and yields.

Source / platform	Execution date	Search strategy (platform-adapted syntax)	Filters	Records
PubMed/MEDLINE	10 June 2026	("descending genicular artery" OR "descending genicular arteries" OR "genicular artery" OR "genicular arteries" OR "genicular artery embolization" OR "genicular artery embolisation" OR "genicular embolization" OR "genicular embolisation") AND (knee OR osteoarthritis OR angiography OR angiographic OR embolization OR embolisation OR "digital subtraction angiography" OR DSA OR "cone-beam CT" OR "cone beam CT" OR CBCT OR anatomy OR anatomical OR cadaveric OR dissection OR variant OR branching OR anastomosis)	None	464
Scopus	10 June 2026	TITLE-ABS-KEY(("descending genicular artery" OR "descending genicular arteries" OR "genicular artery" OR "genicular arteries" OR "genicular artery embolization" OR "genicular artery embolisation" OR "genicular	None	684

Source / platform	Execution date	Search strategy (platform-adapted syntax)	Filters	Records
		embolization" OR "genicular embolisation") AND (knee OR osteoarthritis OR angiography OR angiographic OR embolization OR embolisation OR "digital subtraction angiography" OR DSA OR "cone-beam CT" OR "cone beam CT" OR CBCT OR anatomy OR anatomical OR cadaveric OR dissection OR variant OR branching OR anastomosis))		
Web of Science Core Collection / SCIE	10 June 2026	TS=(("descending genicular artery" OR "descending genicular arteries" OR "genicular artery" OR "genicular arteries" OR "genicular artery embolization" OR "genicular artery embolisation" OR "genicular embolization" OR "genicular embolisation") AND (knee OR osteoarthritis OR angiography OR angiographic OR embolization OR embolisation OR "digital subtraction angiography" OR DSA OR "cone-beam CT" OR "cone beam CT" OR CBCT OR anatomy OR anatomical OR cadaveric OR dissection OR variant OR branching OR anastomosis))	None	546
CENTRAL / Ovid EBM Reviews	10 June 2026	("descending genicular artery" OR "descending genicular arteries" OR "genicular artery embolization" OR "genicular artery embolisation" OR "genicular embolization" OR "genicular	No date, language, full-text, or document-type filters; related terms disabled	461

Source / platform	Execution date	Search strategy (platform-adapted syntax)	Filters	Records
		embolisation" OR ("genicular artery" AND embolization) OR ("genicular artery" AND embolisation) OR ("genicular arteries" AND embolization) OR ("genicular arteries" AND embolisation) OR ("genicular artery" AND angiography) OR ("genicular arteries" AND angiography))		
LILACS / BVS, LILACS Plus collection	10 June 2026	("descending genicular artery" OR "arteria genicular descendente" OR "artéria genicular descendente" OR "genicular artery" OR "genicular arteries" OR "arterias geniculares" OR "artérias geniculares" OR "genicular artery embolization" OR "genicular artery embolisation" OR "embolización de arterias geniculares" OR "embolização de artérias geniculares") AND (anatomia OR anatomy OR cadaver OR cadaveric OR cadáver OR dissection OR disección OR dissecção OR angiography OR angiografía OR angiografia OR embolization OR embolisation OR embolización OR embolização OR anastomosis OR anastomose OR variante OR variant OR ramificación OR ramificação)	LILACS Plus only; no full-text, date, language, or document-type filters	31

Note: LILACS was searched in the LILACS Plus collection rather than the complete BVS collection to avoid double counting MEDLINE and other non-prespecified sources already captured through separate searches.

Targeted update: On 18 August 2026, PubMed/MEDLINE and publisher-indexed records were checked using the core DGA/GAE/anatomy terms. Six recent contextual publications were identified; none changed the prespecified quantitative datasets. Details are presented in Table S10.

Table S2. PRISMA 2020 checklist.

Section/topic	Item	Checklist item	Location
Title	1	Identify the report as a systematic review.	Title page
Abstract	2	Structured summary with objectives, eligibility criteria, information sources, synthesis methods, results, limitations and conclusions.	Abstract
Rationale	3	Rationale in context of existing knowledge.	Introduction
Objectives	4	Explicit objective statement.	End of Introduction
Eligibility criteria	5	Inclusion/exclusion criteria and grouping for synthesis.	Methods 2.2; Methods 2.5
Information sources	6	Databases and dates.	Methods 2.3; Table S1
Search strategy	7	Full database-specific search strategies.	Table S1
Selection process	8	Screening and eligibility procedures.	Methods 2.4
Data collection process	9	Extraction and verification procedures.	Methods 2.5
Data items	10	Outcomes and variables sought.	Methods 2.5; Tables S5 and S7
Risk of bias	11	Quality appraisal methods.	Methods 2.6; Tables S4 and S6
Effect measures	12	Effect measures for outcomes.	Methods 2.7
Synthesis methods	13	Data preparation, synthesis, heterogeneity and sensitivity methods.	Methods 2.7 and 2.9; Results 3.3–3.5 and 3.9; Figures 4–7 and S1–S6; Tables S7–S9
Reporting bias	14	Risk of bias due to missing results.	Not formally assessed; rationale in Methods 2.7 and Discussion
Certainty assessment	15	Certainty/confidence methods.	Quality appraisal; no formal GRADE
Study selection	16	Flow from records to included studies.	Results 3.1; Figure 2; Table 1; Table S3
Study characteristics	17	Characteristics of included studies.	Results 3.2; Table 3; Table S5
Risk of bias in studies	18	Quality assessments.	Tables S4 and S6
Individual study results	19	Summary statistics for studies/outcomes.	Figures 4–7; Figures S1–S6; Tables S5–S9; Supplementary File S1
Results of syntheses	20	Statistical syntheses and heterogeneity.	Results 3.3–3.5 and 3.9; Table 2; Table S8; Figures 4–7 and S1–S6
Reporting biases	21	Assessment of bias due to missing results.	Narrative only; formal assessment not performed due to outcome heterogeneity and small k

Section/topic	Item	Checklist item	Location
Certainty of evidence	22	Certainty/confidence results.	Quality appraisal summary; no formal GRADE
Discussion	23	Interpretation, limitations and implications.	Discussion
Registration and protocol	24	Registration, access and amendments.	Methods 2.1
Support	25	Funding and support.	Funding
Competing interests	26	Competing interests.	Conflicts of Interest
Availability	27	Data, code and materials.	Data Availability Statement; Supplementary File S1

Note: The checklist reflects the final manuscript and supplementary package provided with this submission. Page and line numbers should be updated automatically or manually in the submission system if requested by the journal.

Table S3. Reports not retrieved, full-text exclusions and context/citation-only records.

Record/report	Status	Reason/category	PRISMA handling
Rifaat et al. (1969), post-operative DGA aneurysm presenting as pulsating haemarthrosis	Not retrieved	Full text unavailable after final retrieval attempts; historical case report; non-blocking for synthesis.	Report not retrieved; not used as evidence
Wen (2025), free DGA chimeric/perforator flap without the great saphenous vein	Not retrieved / bibliographic anomaly	Title/DOI mismatch and possible overlap with Zhang 2024; independent extraction could not be verified.	Report not retrieved; not used as evidence
Xuan (1996), medial superior genicular artery flap/related reconstructive report	Not retrieved	Older non-DGA-core genicular reconstructive evidence; full text unavailable.	Report not retrieved; not used as evidence
Zhu (2012), anatomy of the free DGA perforator flap	Not retrieved	Potentially relevant DGA-perforator report; full text unavailable after final attempts.	Report not retrieved; not used as evidence
Chen (2025), genicular artery perforator keystone flap	Not retrieved	Peripheral reconstructive evidence; full text unavailable.	Report not retrieved; not used as evidence
Zuo et al. (2024/2025), preclinical porcine medial femoral condyle flap/DGA-related model	Excluded after full-text assessment	Non-human porcine model; outside the prespecified human evidence base.	Full-text exclusion: non-human data; not used as evidence
Langade (2025), commentary/editorial on GAE/anatomical outcomes	Context/citation-only	Commentary without original primary data.	Context-only screening record; not used as evidence
Shahid (2021), correspondence related to genicular arterial anatomy	Context/citation-only	Correspondence without original primary data.	Context-only screening record; not used as evidence
Sobba et al. (2025), finite-element analysis related to MFC harvest/angiosome safety	Context/citation-only	Finite-element/biomechanical planning evidence, not primary human anatomical extraction.	Context-only screening record; not used as evidence
Zhou et al. (2020), pediatric DGA tendon-flap reconstruction series	Context/reconstructive-only screening record	Pediatric reconstructive record outside the adult/core evidence set; retained only to document the screening disposition.	Context-only screening record; not used as evidence

Note: This table documents bibliographic records retained exclusively for PRISMA study-selection transparency. These entries are not numbered citations and were not used as supporting evidence in the synthesis. Five reports were not retrieved, one non-human report was excluded after full-text assessment, and four records were retained only for screening/contextual classification; none was counted among the 65 primary studies.

Table S4. Quality-appraisal summary by evidence stratum.

Evidence stratum	Appraisal approach	Use in synthesis	Interpretation
Stratum A: cadaveric/direct anatomy	AQUA-adapted anatomical framework	Primary quantitative and narrative synthesis	Selection, methods, definitions, reporting, and applicability.
Stratum B: imaging/procedural evidence	JBI analytical cross-sectional checklist	Separate in vivo visualization/procedural synthesis	Selection, exposure/imaging validity, outcome measurement, and analysis.
Stratum C: safety reports	JBI case-report checklist	Narrative synthesis	Clinical history, diagnostic assessment, intervention, outcomes, and lessons.
Stratum D: reconstructive applications	JBI case-series checklist; JBI case-report checklist where appropriate	Narrative synthesis	Consecutive inclusion, case definition, reporting completeness, and outcomes.
Context/citation-only records	Not appraised as primary evidence	Not included as primary evidence	Context or citation verification only.

Study-level AQUA/JBI judgments are presented in Table S6. Appraisal disagreements were resolved by consensus, and quality judgments informed interpretation rather than automatic exclusion.

Table S5. Bibliographic reconciliation and study-level traceability for the 65 primary studies.

Ref.	Study	Study focus	Evidence stratum	Design / modality	Synthesis role
[1]	Fonkoue (2021)	Anatomical study of the descending genicular artery and implications for image-guided interventions for knee pain	Cadaveric DGA-core	Cadaveric dissection	Cadaveric DGA quantitative-synthesis contributor
[2]	Garcia-Pumarino (2014)	Anatomical variability of descending genicular artery	Cadaveric DGA-core	Cadaveric dissection	Cadaveric DGA quantitative-synthesis contributor
[3]	Yamamoto (2010)	The arterial anatomy of the medial femoral condyle and its clinical implications	Cadaveric MFC/DGA anatomy	Fresh-frozen cadaveric dissection/injection	Cadaveric DGA/SMGA quantitative-synthesis contributor
[4]	Van Dijck (2011)	Arterial anatomy of the free vascularised corticoperiosteal graft from the medial femoral condyle	Cadaveric MFC/DGA anatomy	Cadaveric dissection	Cadaveric dominance/presence contributor
[5]	Bagla (2021)	Angiographic analysis of the anatomical variants in genicular artery embolization	Angiographic/procedural GAE anatomy	DSA during GAE	Angiographic DGA/target/anastomosis contributor
[6]	Callese (2023)	Classification of genicular artery anatomic variants using intraoperative cone-beam computed tomography	CBCT/GAE anatomical imaging	Cone-beam CT during GAE	Imaging DGA visualization contributor
[7]	Cusumano (2023)	Added value of cone-beam CT to identify arterial supply during GAE	CBCT + DSA procedural imaging	CBCT and DSA during GAE	Modality comparison only
[8]	Taheri Amin (2025)	Multicentric angiographic assessment of the branching patterns and anastomotic network of the genicular arteries	DSA/GAE anatomy	Digital subtraction angiography	Angiographic quantitative-synthesis contributor
[9]	Behzadi (2025)	Evaluating the association between knee osteoarthritis severity and descending genicular artery caliber	CTA vascular physiology	CTA	DGA caliber/OA severity narrative or synthesis
[10]	Fukumoto (2021)	Characteristics of the descending genicular artery blood flow velocity in patients with knee osteoarthritis	Doppler ultrasound physiology	Ultrasound Doppler	Physiology only
[13]	Iorio (2011)	The limits of medial femoral condyle corticoperiosteal flaps	Cadaveric periosteal perfusion	Cadaveric angiographic perfusion	Cadaveric morphometry/perfusion contributor
[14]	Iorio (2012)	Cutaneous angiosome territory of the medial femoral condyle osteocutaneous flap	Cadaveric angiosome + clinical	Cadaveric injection + clinical series	Cadaveric angiosome/perforator contributor
[15]	Sananpanich (2013)	Anatomical variations of the saphenous and descending genicular artery perforators	Cadaveric DGA/reconstructive	Cadaveric dissection	Cadaveric DGA/perforator quantitative-synthesis contributor
[16]	Silva (2018)	Medial femoral condyle corticoperiosteal flap: anatomic study	Cadaveric DGA/MFC anatomy	Cadaveric dissection	Cadaveric DGA quantitative-synthesis contributor
[17]	Weitgasser (2016)	The detailed vascular anatomy of the medial femoral condyle and its significance for its use as a free flap	Cadaveric MFC/DGA anatomy	Thiel cadaveric dissection	Cadaveric DGA branching/SA contributor

Ref.	Study	Study focus	Evidence stratum	Design / modality	Synthesis role
[18]	Kim (2026)	Chimeric descending genicular artery flap: expanding applications of the medial femoral condyle composite flap reconstruction	CTA anatomical applied DGA flap	CTA + clinical/reconstructive	Imaging DGA/SA/perforator contributor
[33]	Tozzi (1996)	Pseudoaneurysm of the descending genicular artery after arthroscopic meniscectomy	DGA pseudoaneurysm case report	CT + angiography	No MA
[34]	Tsubosaka (2017)	Pseudoaneurysm of the articular branch of the descending genicular artery following ACL reconstruction	DGA articular branch pseudoaneurysm	CT + Doppler + embolization	No MA
[35]	Silvestri (2018)	Descending genicular artery injury following transient lateral patellar dislocation	DGA injury case report	CT angiography + embolization	No MA
[36]	Oláh (2025)	Descending genicular artery embolization to reduce chronic pain after total knee replacement	GAE case report post-TKA pain	Angiography/embolization case report	No MA
[19]	O'Grady (2022)	Cadaveric and angiographic anatomical considerations in the genicular arterial system	Cadaveric + angiographic correlation	Cadaveric dissection + angiography	Use cadaveric and angiographic components separately
[20]	Kawarai (2018)	Anatomical features of the descending genicular artery to facilitate surgical exposure for the subvastus approach	Cadaveric DGA-core	Cadaveric dissection	Cadaveric DGA quantitative-synthesis contributor
[21]	Kohlhauser (2025)	An analysis of the medial femoral condyle flap anatomy and the involvement of different tissue components	Cadaveric combined flap anatomy	Thiel cadaveric dissection	Cadaveric DGA branching/SA contributor
[22]	Zhang (2024)	Clinical applications and cadaveric study of the free descending genicular artery perforator flap without the saphenous vein	Cadaveric + clinical reconstructive	Cadaveric dissection + clinical series	Only repeated DGAP-SV variables if comparable
[23]	Mohan (2019)	Reappraisal of perforasomes of the superficial femoral, descending genicular, and saphenous arteries	Perforasome/reconstructive anatomy	Cadaveric CTA + clinical series	Perforator origin/perforasome subgroup only
[24]	Nakatsuka (2026)	Vastus medialis branch of the descending genicular artery as a recipient vessel	Cadaveric + clinical DGA recipient anatomy	Cadaveric dissection + clinical application	Cadaveric branch-specific contributor
[25]	Rahmanian-Schwarz (2011)	Composite osteomusculocutaneous free flap from the medial femoral condyle	Cadaveric + clinical MFC/DGA anatomy	Cadaveric dissection + clinical cases	Cadaveric DGA contributor once only
[26]	Zheng (2015)	Revisit of the anatomy of the distal perforator of the descending genicular artery and clinical application	Cadaveric + clinical DGA perforator	Cadaveric dissection + clinical cases	DGA perforator subgroup contributor
[27]	Kobayashi (2022)	Descending genicular artery as recipient vessel in severe knee trauma reconstruction	DGA recipient vessel case series	Case series	No MA

Ref.	Study	Study focus	Evidence stratum	Design / modality	Synthesis role
[28]	Li (2023)	Free ALT flap using descending genicular artery as recipient vessel around the knee	DGA recipient vessel clinical series	Clinical series	No MA principal; descriptive counts only
[29]	Venkatramani (2014)	Use of descending genicular vessels as recipient vessels for free flaps around the knee	DGA recipient vessel clinical series	Clinical series	No MA
[45]	Xu (2020)	Anatomical study of the descending genicular artery chimeric flaps	Cadaveric DGA/reconstructive	Cadaveric dissection	Cadaveric DGA/perforator quantitative-synthesis contributor
[43]	Başarir (2006)	Safe zone for the descending genicular artery in the midvastus approach to the knee	Cadaveric surgical landmark anatomy	Cadaveric dissection	Landmark/morphometry only
[44]	Thiengwittayaporn (2009)	Anatomical landmark of descending genicular artery for minimidvastus approach of the knee	Cadaveric surgical landmark anatomy	Cadaveric dissection	Landmark/morphometry only
[46]	Andrade (2009)	Anatomical study of the medial femoral condyle flap / DGA-related MFC anatomy	Cadaveric MFC/DGA anatomy	Cadaveric dissection	Cadaveric DGA quantitative-synthesis contributor
[47]	Huang (2011)	An anatomic and clinical study of the adductor magnus tendon-descending genicular artery bone flap	Cadaveric + clinical DGA/adductor tendon	Cadaveric dissection + clinical series	Cadaveric DGA quantitative-synthesis contributor
[48]	Sighary (2022)	Cadaveric classification of the genicular arteries, with implications for the interventional radiologist	Cadaveric genicular anatomy	Cadaveric dissection	DGA branching only; not DGA presence
[49]	Tinhofer (2018)	The dermal arteries in the cutaneous angiosome of the descending genicular artery	Dermal microvascular DGA angiosome	HREM microanatomy	No MA; narrative microanatomy
[50]	Romiti (2006)	Importance of the arteriographic anatomy of the descending genicular artery and sural arteries	Arteriographic collateral anatomy	Arteriography	Pathologic angiographic subgroup only
[51]	Hayashi (1990)	The medial genicular artery flap	SMGA/reconstructive anatomy	Cadaveric dissection + clinical cases	Non-DGA genicular subgroup only
[52]	Hayashi (1990)	The lateral genicular artery flap	SLGA/reconstructive anatomy	Cadaveric dissection + clinical cases	Non-DGA genicular subgroup only
[53]	Blagojević (2015)	Anatomy of the middle genicular artery	Middle genicular artery anatomy	Cadaveric dissection	Non-DGA MGA subgroup only
[54]	Hirtler (2016)	The inferior medial genicular artery and its vascularization of the pes anserinus superficialis	IMGA/pes anserinus anatomy	Cadaveric dissection	Non-DGA IMGA subgroup only
[55]	Hsieh (2023)	Descending genicular artery perforator flap for defects around the knee	DGAP flap clinical series	Clinical series	No MA
[56]	Christen (2022)	Medial femoral condyle free flap clinical series	MFC free flap clinical series	Clinical series	No MA

Ref.	Study	Study focus	Evidence stratum	Design / modality	Synthesis role
[57]	Guzzini (2022)	Donor site regeneration following medial femoral condyle flap harvest	MFC donor-site safety	Clinical series + MRA subset	No MA
[58]	Guzzini (2016)	Treatment of femur nonunion with microsurgical corticoperiosteal pedicled flap from MFC	Reconstructive case report	Case report	No MA
[59]	Grant (2005)	Vascularised bone graft from the medial femoral condyle for failed arthrodesis	Reconstructive case report	Case report	No MA
[60]	Oh (2019)	Intraoperative anatomy of the medial femoral condyle flap / MFC vascular pedicle dominance	Clinical intraoperative MFC anatomy	Clinical intraoperative series	Clinical intraoperative subgroup only
[61]	Pelzer (2010)	Osteo-periosteal-cutaneous flaps of the medial femoral condyle	MFC osteoperiosteal-cutaneous flap	Clinical series	No MA
[62]	Mattos (2019)	Vascularized medial femoral condyle flap for wrist osteomyelitis	MFC flap clinical application	Clinical series	No MA
[63]	Kumta (2017)	Vascularized medial femoral condyle corticoperiosteal graft for nonunion	MFC graft clinical application	Clinical series	No MA
[64]	Low (2022)	Superior lateral genicular artery flap/reconstructive application	SLGA flap context	Clinical/reconstructive	No MA
[37]	Lyrtzis (2025)	Intravascular papillary endothelial hyperplasia involving musculoarticular branch of DGA	DGA branch pathology case report	Case report	No MA
[65]	Park (2001)	Use of descending genicular and superior medial genicular vessels as recipient vessels for free flap reconstruction around the knee	DGA/SMGA recipient vessels	Clinical series	No MA
[66]	Rees-Lee (2011)	Superior medial genicular vessels as recipient vessels for free flap knee reconstruction	SMGA recipient vessel	Clinical series	No MA
[67]	Shen (2012)	Reverse sartorius flap based on medial genicular/pes anserinus anastomotic network	Medial genicular network reconstruction	Clinical series	No MA
[68]	Shirota (2024)	SCIP flap using descending genicular artery as recipient vessel for patellar defect	DGA recipient vessel case report	Case report	No MA
[69]	Taniguchi (2009)	Superior lateral genicular artery flap after total knee arthroplasty	SLGA flap clinical case	Case report	No MA
[70]	Salaria (2008)	Anatomic study of the middle genicular artery	Middle genicular artery anatomy	Cadaveric dissection	Non-DGA MGA subgroup only
[71]	Shahid (2015)	A cadaveric study of branching pattern and diameter of the genicular arteries: focus on middle genicular artery	Genicular arteries/MGA anatomy	Cadaveric dissection	Non-DGA genicular subgroup only

Ref.	Study	Study focus	Evidence stratum	Design / modality	Synthesis role
[72]	Zhang (2018)	DGA-saphenous artery perforator flap for deep burn wounds in elderly patients	DGA-SA perforator flap clinical series	Clinical series	No MA
[73]	Xu (2023)	Superior lateral genicular artery perforator propeller flap	SLGA perforator anatomy/clinical	Cadaveric dissection + clinical cases	Non-DGA SLGA subgroup only
[74]	Yang (2021)	Superior lateral genicular artery perforator propeller flap around the knee	SLGA perforator clinical series	Clinical series	No MA
[75]	Yalçın (2025)	Inferior medial genicular artery based tibia osteocutaneous flap	IMGA osteocutaneous flap anatomy/clinical	Cadaveric dissection + clinical cases	Non-DGA IMGA subgroup only

Note: All 65 rows represent primary studies retained in the qualitative evidence synthesis/evidence map. The table provides a one-to-one crosswalk between study, evidence stratum, synthesis role, and the definitive reference number used in the main manuscript. Methodological references [39–42] and post-cutoff contextual references [11,12,30–32,38] are outside this primary-study denominator.

Table S6. Study-level methodological quality appraisal of the 65 primary studies.

Study	Appraisal tool	R1 / R2	Consensus	Key concern / message	Use in synthesis
A001: Fonkoue 2021	AQUA-adapted anatomical appraisal	Low concern / Low concern	Low concern	Detailed fresh/fresh-frozen cadaveric DGA-focused anatomy with clear n/N and morphometry.	Yes; Main analysis
A002: Garcia-Pumarino 2014	AQUA-adapted anatomical appraisal	Some concern / Some concern	Some concern	Useful DGA anatomy; some missing dispersion data and harmonization decisions needed for DGA trunk definition.	Yes; sensitivity; Include main; sensitivity for DGA presence definition
A003: Kawarai 2018	AQUA-adapted anatomical appraisal	Low concern / Low concern	Low concern	Large cadaveric knee sample with explicit DGA presence/absence and branching types; embalming may affect dimensions.	Yes; Main analysis
A004: O'Grady 2022	AQUA-adapted anatomical appraisal	Low concern / Low concern	Low concern	Cadaveric component is clear and DGA-specific; angiographic component must remain separated from cadaveric pooling.	Yes; cadaveric component only; Main analysis
A005: Sighary 2022	AQUA-adapted anatomical appraisal	Some concern / Some concern	Some concern	Excellent classification study, but denominator relates to preserved/reviewed genicular arteries rather than DGA presence in all limbs.	No for DGA presence; yes for branching narrative; Exclude from DGA-presence MA
A006: Sananpanich 2013	AQUA-adapted anatomical appraisal	Low concern / Low concern	Low concern	Fresh cadaveric reconstructive anatomy with extractable DGA/SA/perforator n/N.	Yes; Main analysis
A007: Xu 2020	AQUA-adapted anatomical appraisal	Some concern / Some concern	Some concern	Small fresh cadaveric series; useful DGA/SA/OAB data but limited precision.	Yes; sensitivity; Include main; sensitivity for small sample
A008: Zhang 2024	AQUA-adapted anatomical appraisal	Some concern / Some concern	Some concern	Cadaveric component useful; clinical component includes pediatric cases and must not be mixed with adult anatomical inference.	Yes; cadaveric variables only; Use cadaveric component only
A009: Thiengwittayaporn 2009	AQUA-adapted anatomical appraisal	Some concern / Some concern	Some concern	Landmark-focused study; useful for surgical safe-zone narrative, not for most global anatomical pools.	No for global MA; Narrative/landmark table

Study	Appraisal tool	R1 / R2	Consensus	Key concern / message	Use in synthesis
A010: Başarir 2006	AQUA-adapted anatomical appraisal	Some concern / Some concern	Some concern	Landmark-focused formalin cadaveric study; definitions and reference points differ from DGA-origin studies.	No for global MA; Narrative/landmark table
A011: Iorio 2011	AQUA-adapted anatomical appraisal	Low concern / Low concern	Low concern	Strong DGA periosteal perfusion study with angiographic subtraction and clear morphometry.	Yes; Main analysis
A012: Iorio 2012	AQUA-adapted anatomical appraisal	Low concern / Low concern	Low concern	Strong DGA/SAB cutaneous angiosome study; clinical series should be kept separate from cadaveric component.	Yes; relevant variables only; Main analysis; monitor Iorio-group overlap
A013: Silva 2018	AQUA-adapted anatomical appraisal	Low concern / Low concern	Low concern	Clear fresh-cadaveric MFC/DGA study with DGA presence, branch origin, length, and diameter.	Yes; Main analysis
A014: Yamamoto 2010	AQUA-adapted anatomical appraisal	Low concern / Low concern	Low concern	High-value fresh-frozen cadaveric arterial anatomy with clear DGA/SMGA branch data and perforator mapping.	Yes; Main analysis
A015: Weitgasser 2016	AQUA-adapted anatomical appraisal	Low concern / Low concern	Low concern	Large Thiel cadaveric study with explicit vascular patterns; possible institutional overlap with Kohlhauser must be controlled by variable.	Yes; overlap-aware; Main analysis; overlap check
A016: Van Dijck 2011	AQUA-adapted anatomical appraisal	Some concern / Some concern	Some concern	Valuable dominance and DGA/SMGA data; phenol preservation and dominance framing require careful harmonization.	Yes; sensitivity; Include with sensitivity
A017: Kohlhauser 2025	AQUA-adapted anatomical appraisal	Low concern / Low concern	Low concern	Recent Thiel cadaveric study with rich branch-level data; possible Graz overlap with Weitgasser controlled by variable.	Yes; overlap-aware; Main analysis; overlap check
A018: Andrade 2009	AQUA-adapted anatomical appraisal	Some concern / Some concern	Some concern	Small anatomical series with useful DGA measurements; reporting detail moderate.	Yes; sensitivity; Include with sensitivity
A019: Huang 2011	AQUA-adapted anatomical appraisal	Low concern / Low concern	Low concern	Detailed cadaveric + clinical DGA tendon flap study; anatomical component extractable.	Yes; anatomical component; Main analysis
A020: Nakatsuka 2026	AQUA-adapted anatomical appraisal	Low concern / Low concern	Low concern	Recent cadaveric branch-specific study with DGA and vastus medialis branch data; some dispersion data incomplete.	Yes; branch-specific; Main/secondary analysis
A021: Rahmanian-Schwarz 2011	AQUA-adapted anatomical appraisal	Some concern / Some concern	Some concern	The report was treated as a single study after bibliographic deduplication; one contribution was permitted per eligible outcome.	Yes; one contribution per eligible outcome
A022: Zheng 2015	AQUA-adapted anatomical appraisal	Low concern / Low concern	Low concern	High-value DGA/OAB perforator cadaveric study with clinical application; perforator definition is subdomain-specific.	Yes; perforator subdomain; Secondary/perforator analysis
X001: Hayashi 1990 medial	AQUA-adapted anatomical appraisal	Some concern / Some concern	Some concern	Older small SMGA flap anatomy; complementary medial network only.	No for DGA MA; Complementary narrative

Study	Appraisal tool	R1 / R2	Consensus	Key concern / message	Use in synthesis
X002: Hayashi 1990 lateral	AQUA-adapted anatomical appraisal	Some concern / Some concern	Some concern	Older small SLGA flap anatomy; complementary lateral network only.	No for DGA MA; Complementary narrative
X003: Blagojević 2015	AQUA-adapted anatomical appraisal	Some concern / Some concern	Some concern	MGA-focused cadaveric anatomy; useful for network context, not DGA-core.	No for DGA MA; Complementary narrative
X004: Hirtler 2016	AQUA-adapted anatomical appraisal	Some concern / Some concern	Some concern	Inferomedial/pes anserinus anatomy; complementary genicular network, not DGA-core.	No for DGA MA; Complementary narrative
X005: Shahid 2015	AQUA-adapted anatomical appraisal	Some concern / Some concern	Some concern	Cadaveric genicular network study focused on popliteal branches/MGA; not DGA-core.	No for DGA MA; Complementary narrative
X006: Salaria 2008	AQUA-adapted anatomical appraisal	Some concern / Some concern	Some concern	Small MGA-focused study; relevant to posterior/intra-articular vascular safety only.	No for DGA MA; Complementary narrative
X008: Tinhofer 2018	AQUA-adapted anatomical appraisal	Low concern / Low concern	Low concern	Excellent microanatomical DGA angiosome/HREM study; not directly comparable with macrovascular DGA metrics.	No for macrovascular MA; Narrative microanatomy
B001: Bagla 2021	JBI analytical cross-sectional / imaging appraisal	Some concern / Some concern	Some concern	Retrospective angiographic analysis from clinical trial procedures; high relevance but small sample and procedural selection.	Yes; angiographic stratum only; Imaging stratum only
B002: Callese 2023	JBI analytical cross-sectional / imaging appraisal	Low concern / Low concern	Low concern	Large CBCT GAE anatomy cohort with clear vessel classification; use as primary CBCT visualization study.	Yes; in vivo visualization stratum; Imaging stratum only
B003: Cusumano 2023	JBI analytical cross-sectional / imaging appraisal	Low concern / Low concern	Low concern	Same UCLA cohort as Callese but different target-identification outcome; do not duplicate DGA presence with Callese.	No for duplicated DGA presence; yes for modality comparison; Imaging stratum only
B004: Taheri Amin 2025	JBI analytical cross-sectional / imaging appraisal	Low concern / Low concern	Low concern	Large multicenter DSA/GAE anastomosis evidence; useful for angiographic anastomosis and procedural mapping.	Yes; angiographic stratum; Imaging stratum only
B005: Behzadi 2025	JBI analytical cross-sectional / imaging appraisal	Low concern / Low concern	Low concern	CTA cross-sectional physiology; strong measurement approach but not anatomical prevalence.	No for prevalence; narrative/physiology and possible continuous association; Imaging stratum only
B006: Fukumoto 2021	JBI analytical cross-sectional / imaging appraisal	Low concern / Low concern	Low concern	Doppler physiology with control group and reproducibility; small sample and not anatomical prevalence.	No for prevalence; physiology narrative; Imaging stratum only
B007: Romiti 2006	JBI analytical cross-sectional / imaging appraisal	Some concern / Some concern	Some concern	Pathologic arteriographic collateral cohort; external validity limited for OA/GAE anatomy.	No for OA/GAE pools; narrative collateral anatomy; Imaging stratum only
B008: Kim 2026	JBI analytical cross-sectional / imaging appraisal	Some concern / Some concern	Some concern	CTA planning study with useful DGA-axis data; not cadaveric prevalence and may involve selected imaging population.	Conditional; imaging stratum only; Imaging stratum only

Study	Appraisal tool	R1 / R2	Consensus	Key concern / message	Use in synthesis
D001: Hsieh 2023	JBI case-series appraisal	Some concern / Some concern	Some concern	Small DGAP clinical series; reconstructive relevance high but low quantitative generalizability.	Yes; Narrative/reconstructive
D002: Christen 2022	JBI case-series appraisal	Some concern / Some concern	Some concern	Small MFC free flap series; useful clinical context only.	Yes; Narrative/reconstructive
D003: Guzzini 2022	JBI case-series appraisal	Low concern / Low concern	Low concern	Larger donor-site outcome series; strong for reconstructive safety but not anatomical pooling.	Yes; Narrative/reconstructive
D004: Guzzini 2016	JBI case-report appraisal	Some concern / Some concern	Some concern	MFC pedicled flap case report; reconstructive proof-of-concept.	Narrative only; Case report
D005: Grant 2005	JBI case-report appraisal	Some concern / Some concern	Some concern	Historical vascularized MFC graft case; reconstructive context.	Narrative only; Case report
D006: Kobayashi 2022	JBI case-series appraisal	Some concern / Some concern	Some concern	Three-case recipient-vessel series; useful narrative evidence only.	Yes; Narrative/reconstructive
D007: Kumta 2017	JBI case-series appraisal	Some concern / Some concern	Some concern	Clinical MFC graft series; reconstructive context, not DGA anatomy.	Yes; Narrative/reconstructive
D008: Li 2023	JBI case-series appraisal	Some concern / Some concern	Some concern	DGA/branch recipient vessel series for ALT flaps; clear technique but small sample.	Yes; Narrative/reconstructive
D009: Low 2022	JBI case-series appraisal	Some concern / Some concern	Some concern	SLGA complementary network series; not DGA-core.	Yes; Narrative/reconstructive
D010: Mattos 2019	JBI case-series appraisal	Some concern / Some concern	Some concern	Small MFC flap clinical series for wrist osteomyelitis; useful narrative.	Yes; Narrative/reconstructive
D011: Oh 2019	JBI case-series appraisal	Low concern / Low concern	Low concern	Large intraoperative MFC/MFT series; valuable for clinical anatomy translation.	Yes; clinical anatomy table; Narrative/reconstructive
D012: Pelzer 2010	JBI case-series appraisal	Some concern / Some concern	Some concern	Osteoperiosteal-cutaneous MFC flaps, small series.	Yes; Narrative/reconstructive
D013: Park 2001	JBI case-series appraisal	Some concern / Some concern	Some concern	Historical recipient-vessel series around knee; technique and outcomes clear but small.	Yes; Narrative/reconstructive
D014: Rees-Lee 2011	JBI case-series appraisal	Some concern / Some concern	Some concern	SMGA recipient-vessel series; complementary non-DGA evidence.	Yes; Narrative/reconstructive
D015: Shen 2012	JBI case-series appraisal	Some concern / Some concern	Some concern	Retrograde sartorius flap series; useful medial network context.	Yes; Narrative/reconstructive
D016: Shirota 2024	JBI case-report appraisal	Low concern / Low concern	Low concern	DGA can serve as recipient vessel for SCIP flap around patella in selected cases.	Narrative only; Case report
D017: Taniguchi 2009	JBI case-report appraisal	Some concern / Some concern	Some concern	SLGA flap case after TKA; complementary lateral network safety/reconstruction.	Narrative only; Case report
D018: Venkatramani 2014	JBI case-series appraisal	Some concern / Some concern	Some concern	Free flap series using DGA recipient vessels; relevant narrative only.	Yes; Narrative/reconstructive

Study	Appraisal tool	R1 / R2	Consensus	Key concern / message	Use in synthesis
D019: Zhang 2018	JBI case-series appraisal	Some concern / Some concern	Some concern	Large clinical DGA-saphenous perforator flap series; outcomes strong but anatomical details not sufficient for pooling.	Yes; Narrative/reconstructive
D020: Xu 2023	JBI case-series appraisal	Some concern / Some concern	Some concern	SLGA propeller anatomy/clinical; complementary lateral network.	Yes; Narrative/reconstructive
D021: Yang 2021	JBI case-series appraisal	Some concern / Some concern	Some concern	SLGA perforator propeller clinical series; complementary non-DGA.	Yes; Narrative/reconstructive
D022: Yalçın 2025	JBI case-series appraisal	Some concern / Some concern	Some concern	IMGA-based osteocutaneous flap; complementary inferior-medial network.	Yes; Narrative/reconstructive
X007: Mohan 2019	JBI case-series appraisal	Low concern / Low concern	Low concern	High-quality perforasome study combining cadaveric CT angiography and clinical cases; not DGA-core presence.	Yes; perforasome narrative; Narrative/reconstructive
C001: Oláh 2025	JBI case-report appraisal	Low concern / Low concern	Low concern	DGA embolization can be relevant for selected post-TKA pain with focal hypervascularity.	Narrative only; Case report
C002: Silvestri 2018	JBI case-report appraisal	Low concern / Low concern	Low concern	DGA branches are vulnerable after patellar dislocation; CT/angiography and embolization can be decisive.	Narrative only; Case report
C003: Tozzi 1996	JBI case-report appraisal	Some concern / Some concern	Some concern	Historical DGA pseudoaneurysm after arthroscopy; useful but reporting standards older.	Narrative only; Case report
C004: Tsubosaka 2017	JBI case-report appraisal	Low concern / Low concern	Low concern	Articular branch DGA pseudoaneurysm can follow far anteromedial ACL portal; embolization is effective.	Narrative only; Case report
C005: Lyrtzis 2025	JBI case-report appraisal	Some concern / Some concern	Some concern	Rare DGA branch pathology; useful narrative only.	Narrative only; Case report

Table S7. Cadaveric unit-of-analysis audit.

Study	Citation	Cadavers	Anatomical units	Units/cadaver	Status	Sensitivity rule
Fonkoue 2021	1	21	27	1.29	Known clustered/bilateral	Design effect m at ρ = 1
Garcia-Pumarino 2014	2	20	20	1.00	Known one unit per cadaver	Design effect m at ρ = 1
Kawarai 2018	20	NR	70	NR	Cadaver count unavailable	Conservative m = 2 at ρ = 1
O'Grady 2022	19	20	20	1.00	Known one unit per cadaver	Design effect m at ρ = 1
Sananpanich 2013	15	16	31	1.94	Known clustered/bilateral	Design effect m at ρ = 1
Xu 2020	45	10	15	1.50	Known clustered/bilateral	Design effect m at ρ = 1
Zhang 2024	22	NR	15	NR	Cadaver count unavailable	Conservative m = 2 at ρ = 1
Thiengwittayaporn 2009	44	24	48	2.00	Known clustered/bilateral	Design effect m at ρ = 1
Başarir 2006	43	15	30	2.00	Known clustered/bilateral	Design effect m at ρ = 1

Study	Citation	Cadavers	Anatomical units	Units/cadaver	Status	Sensitivity rule
Iorio 2012	14	12	12	1.00	Known one unit per cadaver	Design effect m at ρ = 1
Iorio 2011	13	18	18	1.00	Known one unit per cadaver	Design effect m at ρ = 1
Silva 2018	16	20	30	1.50	Known clustered/bilateral	Design effect m at ρ = 1
Yamamoto 2010	3	NR	19	NR	Cadaver count unavailable	Conservative m = 2 at ρ = 1
Weitgasser 2016	17	25	50	2.00	Known clustered/bilateral	Design effect m at ρ = 1
Van Dijck 2011	4	27	34	1.26	Known clustered/bilateral	Design effect m at ρ = 1
Kohlhauser 2025	21	NR	35	NR	Cadaver count unavailable	Conservative m = 2 at ρ = 1
Andrade 2009	46	8	15	1.88	Known clustered/bilateral	Design effect m at ρ = 1
Huang 2011	47	NR	34	NR	Cadaver count unavailable	Conservative m = 2 at ρ = 1
Nakatsuka 2026	24	15	30	2.00	Known clustered/bilateral	Design effect m at ρ = 1
Rahmanian-Schwarz 2011	25	11	21	1.91	Known clustered/bilateral	Design effect m at ρ = 1
Zheng 2015	26	NR	40	NR	Cadaver count unavailable	Conservative m = 2 at ρ = 1

Table S8. Unit-of-analysis sensitivity results.

Dataset	Analysis	k	Estimate	95% CI	I²	τ²
PROP DGA PRESENCE STRICT	Original unit-level	16	0.916	0.866–0.948	30.3%	0.246
PROP DGA PRESENCE STRICT	Worst-case clustered (ρ = 1; unknown m = 2)	16	0.897	0.847–0.932	0.0%	0.000
PROP DGA PRESENCE STRICT	Known cadaver count only (ρ = 1)	10	0.888	0.809–0.936	1.9%	0.016
PROP DGA PRESENCE MAIN	Original unit-level	21	0.933	0.896–0.958	37.3%	0.407
PROP DGA PRESENCE MAIN	Worst-case clustered (ρ = 1; unknown m = 2)	21	0.914	0.872–0.943	6.5%	0.076
PROP DGA PRESENCE MAIN	Known cadaver count only (ρ = 1)	15	0.916	0.861–0.951	15.8%	0.201
PROP DGA ORIGIN FEMORAL SFA	Original unit-level	18	0.924	0.892–0.947	12.8%	0.091
PROP DGA ORIGIN FEMORAL SFA	Worst-case clustered (ρ = 1; unknown m = 2)	18	0.919	0.885–0.944	1.9%	0.020
PROP DGA ORIGIN FEMORAL SFA	Known cadaver count only (ρ = 1)	13	0.935	0.891–0.962	16.4%	0.246
PROP DGA ORIGIN POPLITEAL	Original unit-level	17	0.032	0.022–0.046	0.0%	0.000
PROP DGA ORIGIN POPLITEAL	Worst-case clustered (ρ = 1; unknown m = 2)	17	0.030	0.021–0.043	0.0%	0.000
PROP DGA ORIGIN POPLITEAL	Known cadaver count only (ρ = 1)	12	0.022	0.017–0.028	0.0%	0.000
PROP SA FROM DGA BROAD	Original unit-level	15	0.795	0.680–0.876	71.8%	0.706
PROP SA FROM DGA BROAD	Worst-case clustered (ρ = 1; unknown m = 2)	15	0.773	0.657–0.857	55.2%	0.519
PROP SA FROM DGA BROAD	Known cadaver count only (ρ = 1)	10	0.716	0.570–0.828	56.1%	0.413
PROP AB OAB FROM DGA	Original unit-level	14	0.903	0.841–0.942	35.7%	0.291

Dataset	Analysis	k	Estimate	95% CI	I²	τ²
PROP AB OAB FROM DGA	Worst-case clustered (ρ = 1; unknown m = 2)	14	0.880	0.818–0.923	0.0%	0.000
PROP AB OAB FROM DGA	Known cadaver count only (ρ = 1)	9	0.876	0.786–0.931	0.0%	0.000
CONT DGA ORIGIN DISTANCE	Original unit-level	9	13.351	12.824–13.878	80.2%	0.347
CONT DGA ORIGIN DISTANCE	Worst-case clustered (ρ = 1; unknown m = 2)	9	13.343	12.824–13.862	67.3%	0.282
CONT DGA ORIGIN DISTANCE	Known cadaver count only (ρ = 1)	7	13.354	12.797–13.912	57.3%	0.189
CONT DGA DIAMETER	Original unit-level	9	2.073	1.787–2.359	95.2%	0.125
CONT DGA DIAMETER	Worst-case clustered (ρ = 1; unknown m = 2)	9	2.081	1.798–2.364	91.1%	0.113
CONT DGA DIAMETER	Known cadaver count only (ρ = 1)	6	2.207	1.784–2.630	92.7%	0.132

Table S9. Study-level definition of the strict and broad cadaveric DGA-presence datasets.

Study	Strict	Broad	Reason for broad-only inclusion
Fonkoue 2021 [1]	Included	Included	Eligible for the strict unconstrained-presence dataset.
Garcia-Pumarino 2014 [2]	Included	Included	Eligible for the strict unconstrained-presence dataset.
Kawarai 2018 [20]	Included	Included	Eligible for the strict unconstrained-presence dataset.
O'Grady 2022 [19]	Included	Included	Eligible for the strict unconstrained-presence dataset.
Sananpanich 2013 [15]	Included	Included	Eligible for the strict unconstrained-presence dataset.
Xu 2020 [45]	Included	Included	Eligible for the strict unconstrained-presence dataset.
Zhang 2024 [22]	Included	Included	Eligible for the strict unconstrained-presence dataset.
Thiengwittayaporn 2009 [44]	Excluded	Included	Landmark study in which the DGA was identified in all included knees; identification structurally constrained by design.
Başarir 2006 [43]	Excluded	Included	Landmark/safe-zone study in which the DGA was identified in all included knees; identification structurally constrained by design.
Iorio 2012 [14]	Excluded	Included	Flap/angiosome study in which DGA identification was required by the inclusion/design framework.
Iorio 2011 [13]	Excluded	Included	Flap/perfusion study in which DGA identification was required by the inclusion/design framework.
Silva 2018 [16]	Included	Included	Eligible for the strict unconstrained-presence dataset.
Yamamoto 2010 [3]	Included	Included	Eligible for the strict unconstrained-presence dataset.
Weitgasser 2016 [17]	Included	Included	Eligible for the strict unconstrained-presence dataset.
Van Dijck 2011 [4]	Included	Included	Eligible for the strict unconstrained-presence dataset.
Kohlhauser 2025 [21]	Included	Included	Eligible for the strict unconstrained-presence dataset.
Andrade 2009 [46]	Included	Included	Eligible for the strict unconstrained-presence dataset.
Huang 2011 [47]	Included	Included	Eligible for the strict unconstrained-presence dataset.
Nakatsuka 2026 [24]	Included	Included	Eligible for the strict unconstrained-presence dataset.
Rahmanian-Schwarz 2011 [25]	Excluded	Included	Reconstructive/flap study in which the DGA axis was structurally preselected.
Zheng 2015 [26]	Included	Included	Eligible for the strict unconstrained-presence dataset.

Table S10. Targeted recent-literature update performed on 18 August 2026.

Citation key	Title	Source	Evidence type	Disposition in revision
Sajan 2025 [11]	Anatomic Considerations for Osteoarthritis Treatment Strategies	Seminars in Interventional Radiology	Secondary review / contextual update	Discussed as recent anatomical/interventional context; not added to pooled datasets
Brown 2025 [12]	Genicular Artery Embolization: A Technical Review of Anatomy, Pathophysiology, Current Experiences, and Future Directions	Journal of Clinical Medicine	Secondary review / contextual update	Discussed as recent anatomical and technical context; not added to pooled datasets
Balaguruswamy 2026 [30]	Descending Genicular Artery Access for Retrograde Recanalization of a Long-Segment Occlusion of the Superficial Femoral Artery	Journal of Vascular and Interventional Radiology	Technical case / access	Post-search contextual evidence; no eligible DGA anatomical denominator for existing pools
Kalem 2026 [31]	CT angiography-based analysis of femoral artery anatomy for medial distal femoral approaches in children	Injury	CTA anatomy	Post-search pediatric CTA context; not incorporated into adult/cadaveric pooled datasets
Zhong 2026 [32]	Clinical efficacy of ALTF combined with Y-shaped great saphenous vein graft in repairing large soft tissue defects	Chinese Journal of Burns and Wounds	Reconstructive clinical series	Post-search reconstructive context; no change to prespecified quantitative anatomy pools
El Mekhtoume 2026 [38]	Aneurysm of a supra-articular branch of the popliteal artery mimicking a soft-tissue sarcoma	Cureus	Safety case report	Post-search safety context; no change to pooled anatomical estimates

Supplementary File S1. Analysis code and frozen datasets archive (provided separately).

Supplementary Figures

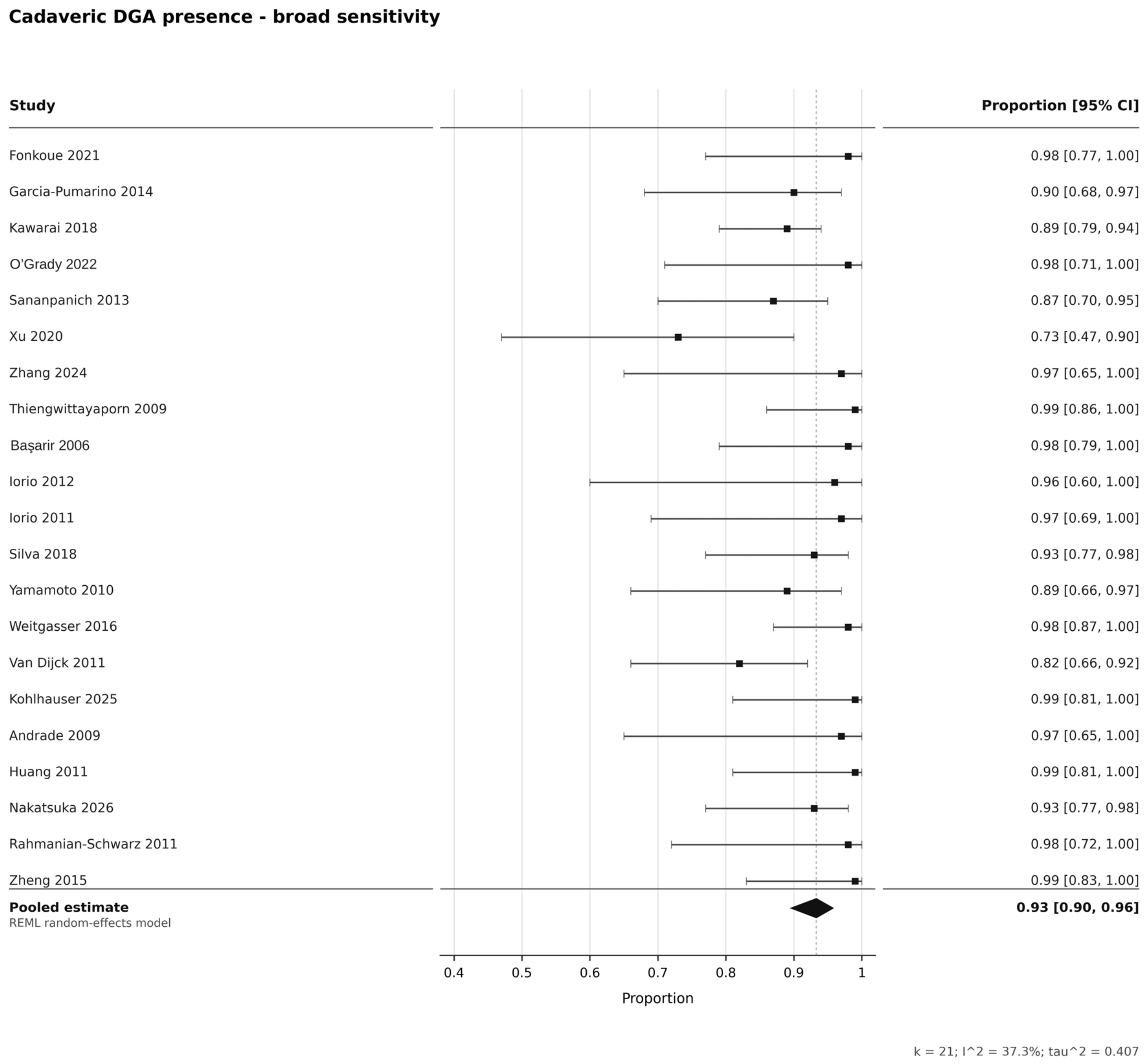

Figure S1. Broad cadaveric DGA presence analysis. This sensitivity analysis includes studies in which DGA identification may have been partially constrained by study design [1–4,13–17,19–22,24–26,43–47].

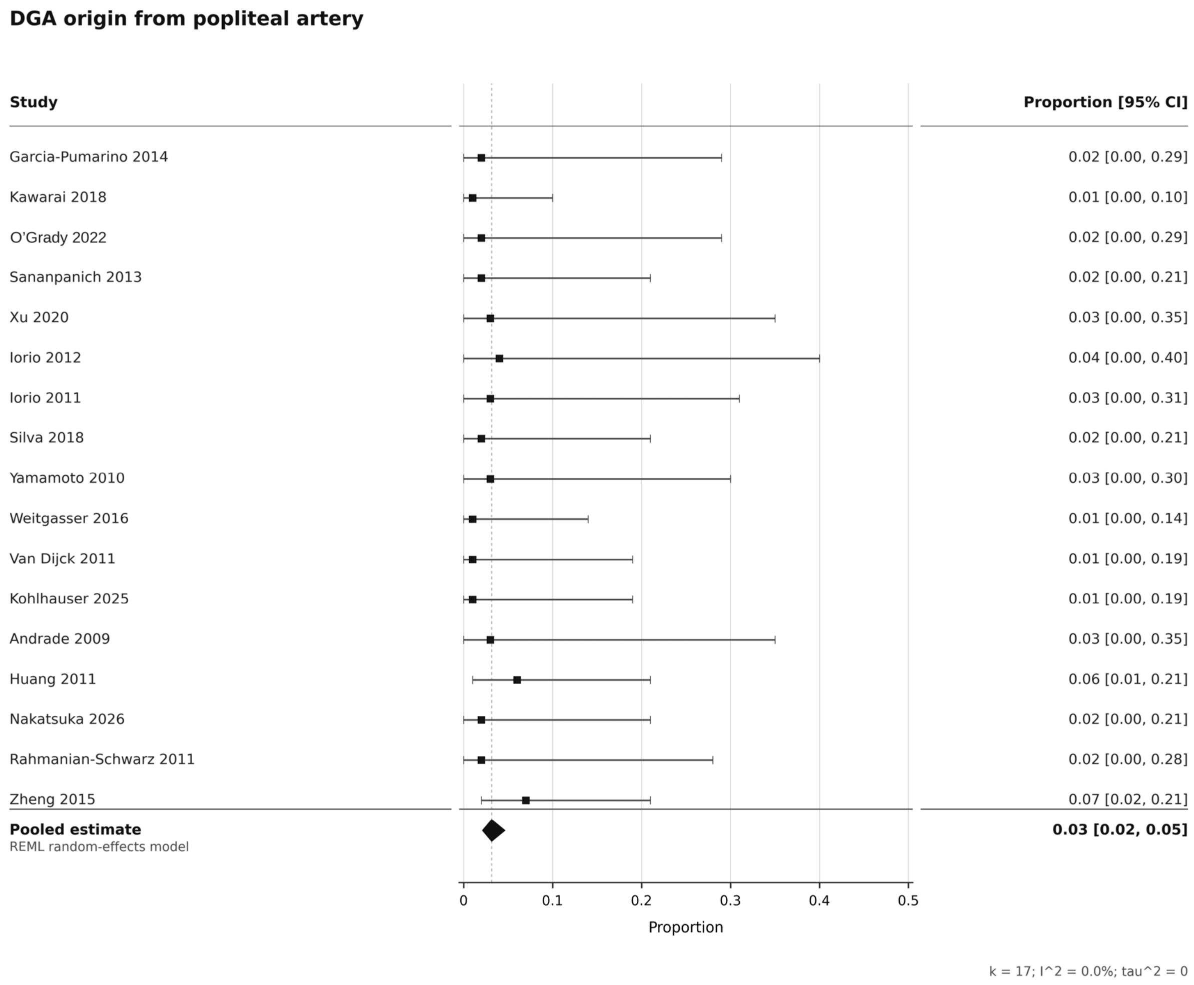

Figure S2. Cadaveric synthesis of DGA origin from the popliteal artery. This rare-variant estimate was retained as supplementary secondary anatomical evidence [2–4,13–17,19–21,24–26,45–47].

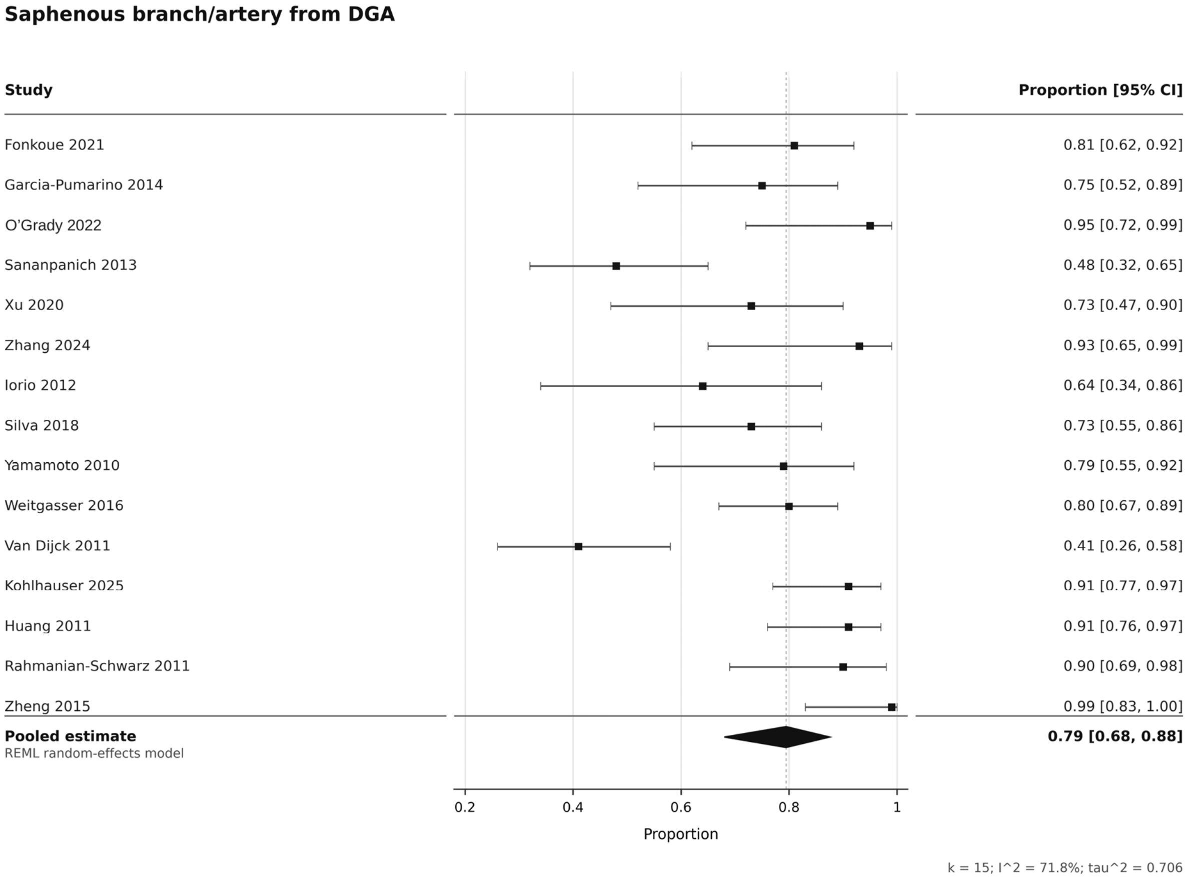

Figure S3. Saphenous branch or saphenous artery arising from the DGA. This synthesis was retained as supplementary because terminology and anatomical definitions varied across studies [1–4,14–17,19,21,22,25,26,45,47].

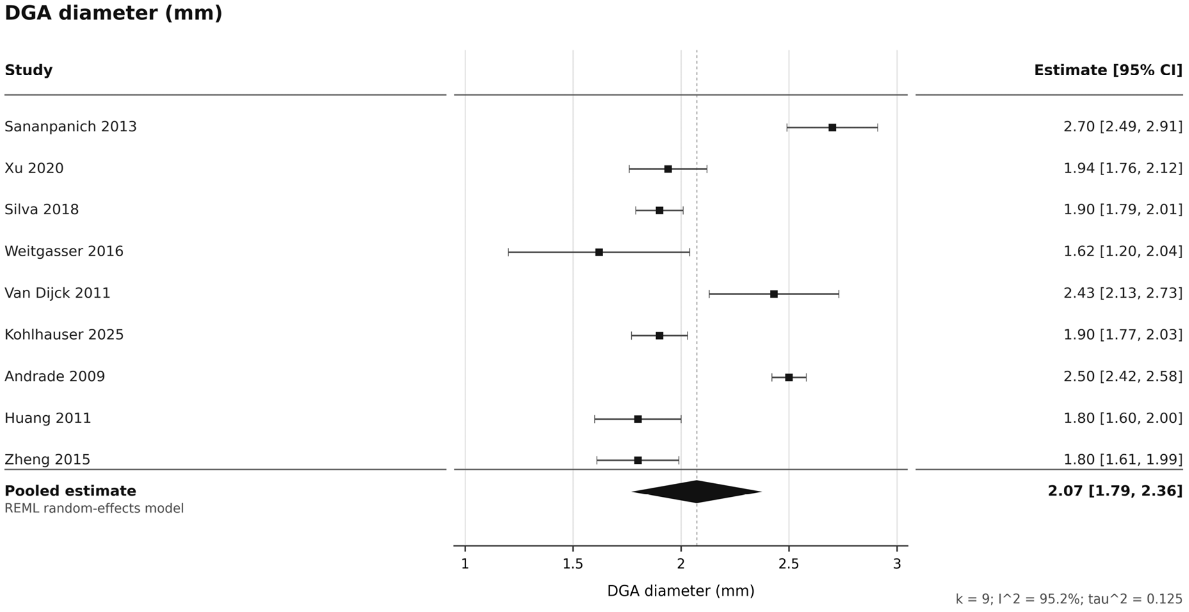

Figure S4. DGA diameter in cadaveric studies. This continuous outcome was retained as exploratory because preservation method, measurement site, and internal versus external diameter reporting varied substantially [4,15–17,21,26,45–47].

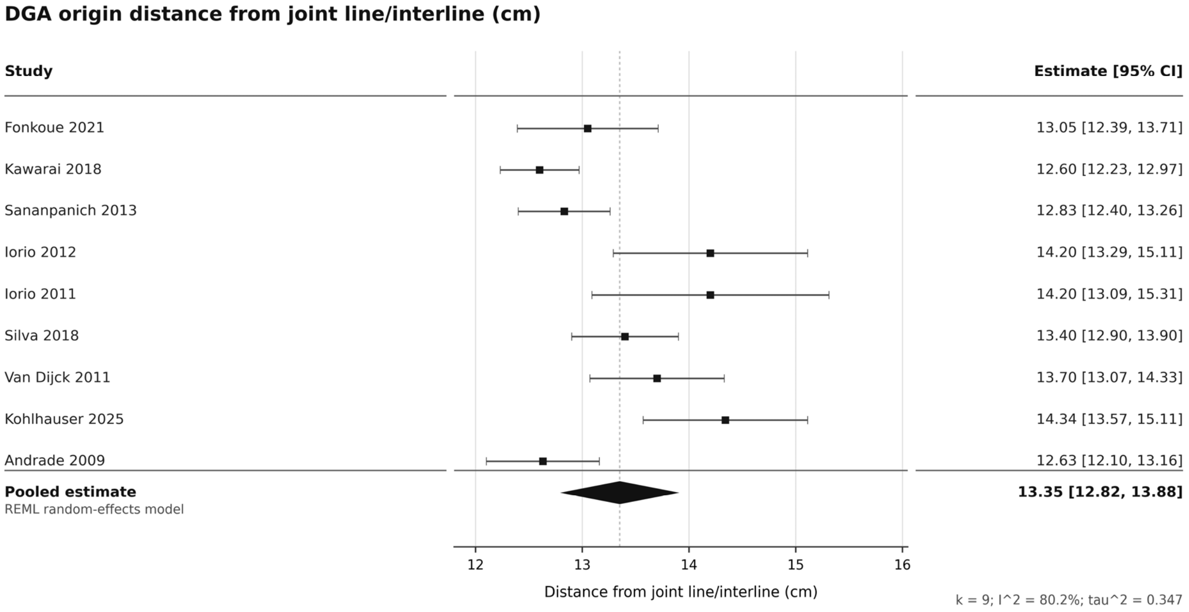

Figure S5. Distance from DGA origin to the knee joint line or interline. This continuous outcome was retained as exploratory because anatomical landmarks and measurement methods were heterogeneous [1,4,13–16,20,21,46].

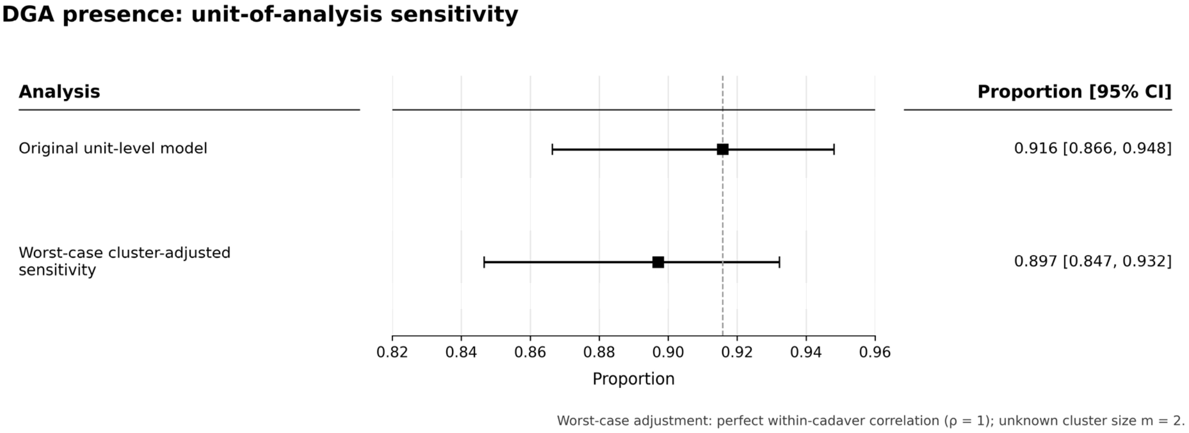

Figure S6. Unit-of-analysis sensitivity for strict cadaveric DGA presence. The worst-case design-effect analysis assumed perfect within-cadaver correlation (ρ = 1) and, when the donor count was unavailable, two anatomical units per cadaver [1–4,15–17,19–22,24,26,45–47].

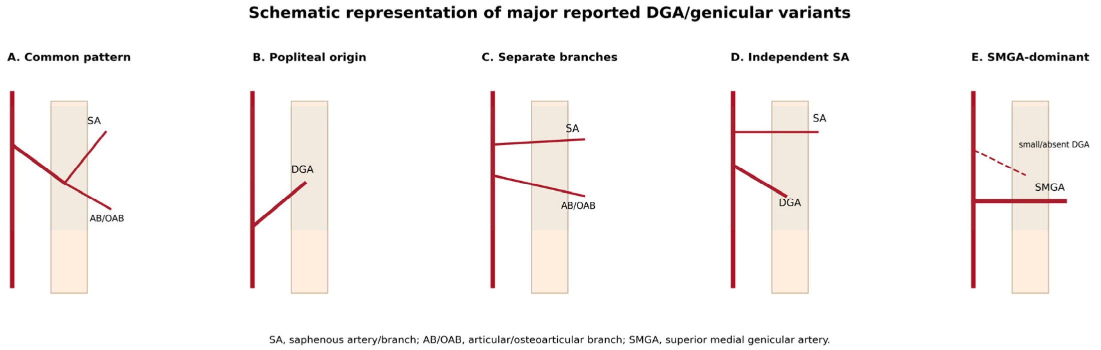

Figure S7. Author-generated schematic of major reported DGA/genicular configurations: common DGA branching, popliteal origin, separate saphenous and articular/osteoarticular branches, an independently arising saphenous artery, and SMGA-dominant supply. The schematic summarizes reported patterns and is not intended to establish a universal classification [1–4,13–17,19–26,43–54].
